# Electrical impedance myography detects age-related skeletal muscle atrophy in adult zebrafish

**DOI:** 10.1038/s41598-023-34119-6

**Published:** 2023-05-03

**Authors:** Seward B. Rutkove, Santiago Callegari, Holly Concepcion, Tyler Mourey, Jeffrey Widrick, Janice A. Nagy, Anjali K. Nath

**Affiliations:** 1 Department of Neurology, Beth Israel Deaconess Medical Center, Boston, MA 02215 USA; 2 Harvard Medical School, Boston, MA 02215 USA; 3 Department of Cardiology, Beth Israel Deaconess Medical Center, Boston, MA 02215 USA; 4 Zebrafish Core Facility, Beth Israel Deaconess Medical Center, Boston, MA 02215 USA; 5 Division of Genetics and Genomics, Boston Children’s Hospital, Boston, MA 02115 USA; 6 Broad Institute, Cambridge, MA 02142 USA

**Keywords:** Neuromuscular disease, Ageing

## Abstract

Age-related deficits in skeletal muscle function, termed sarcopenia, are due to loss of muscle mass and changes in the intrinsic mechanisms underlying contraction. Sarcopenia is associated with falls, functional decline, and mortality. Electrical impedance myography (EIM)—a minimally invasive, rapid electrophysiological tool—can be applied to animals and humans to monitor muscle health, thereby serving as a biomarker in both preclinical and clinical studies. EIM has been successfully employed in several species; however, the application of EIM to the assessment of zebrafish—a model organism amenable to high-throughput experimentation—has not been reported. Here, we demonstrated differences in EIM measures between the skeletal muscles of young (6 months of age) and aged (33 months of age) zebrafish. For example, EIM phase angle and reactance at 2 kHz showed significantly decreased phase angle (5.3 ± 2.1 versus 10.7 ± 1.5°; p = 0.001) and reactance (89.0 ± 3.9 versus 172.2 ± 54.8 ohms; p = 0.007) in aged versus young animals. Total muscle area, in addition to other morphometric features, was also strongly correlated to EIM 2 kHz phase angle across both groups (r = 0.7133, p = 0.01). Moreover, there was a strong correlation between 2 kHz phase angle and established metrics of zebrafish swimming performance, including turn angle, angular velocity, and lateral motion (r = 0.7253, r = 0.7308, r = 0.7857, respectively, p < 0.01 for all). In addition, the technique was shown to have high reproducibility between repeated measurements with a mean percentage difference of 5.34 ± 1.17% for phase angle. These relationships were also confirmed in a separate replication cohort. Together, these findings establish EIM as a fast, sensitive method for quantifying zebrafish muscle function and quality. Moreover, identifying the abnormalities in the bioelectrical properties of sarcopenic zebrafish provides new opportunities to evaluate potential therapeutics for age-related neuromuscular disorders and to interrogate the disease mechanisms of muscle degeneration.

## Introduction

Age-related deficits in skeletal muscle function, termed sarcopenia, are due to both loss of muscle mass and a reduction in the intrinsic force-generating capacity of muscle^1–3^. Sarcopenia is associated with falls, functional decline, frequency of hospital admission, and mortality^4,5^. In addition to adverse outcomes related to mobility, changes in skeletal muscle mass or quality influence cardiometabolic health, in part, because skeletal muscle is responsible for 80% of post-prandial glucose disposal^6,7^. Concordantly, growing evidence demonstrates a link between sarcopenia, myokine signaling, and cardiometabolic outcomes^8–11^, as well as with cognitive function and dementia^12,13^. The impact of sarcopenia-related morbidity and mortality is substantial, and the projected disease burden is growing as the number of Americans aged 65 or older will double to 95 million by 2060^14^.

A challenge in the field is the lack of widely accepted, standardized tools in the clinic for the diagnosis of sarcopenia^15,16^, as well as accurate metrics or biomarkers of disease progression^15,16^. Muscle quality can be determined by magnetic resonance imaging, computed tomography, or histological analysis following needle biopsy. However, imaging modalities are costly and require extensive data processing, and repeated muscle biopsies are invasive and impractical. The ideal diagnostic tool would be minimally invasive, rapid, accurate, and easy to conduct in clinical settings. Such a tool could also be applied to animals in a similar fashion as to humans, so as to serve as a biomarker in both preclinical and clinical studies. Electrical impedance myography (EIM) is a technology that could serve this purpose.

EIM can be performed with two different approaches: surface and needle. Surface EIM is an entirely noninvasive method for skeletal muscle assessment; however, the biophysical properties of the epidermis and subcutaneous fat also influence the measurements^17,18^. On the other hand, in humans, needle EIM is a minimally invasive method with the electrodes in direct contact with the tissue of interest. Therefore, needle EIM enables the acquisition of electrical impedance data from skeletal muscle without the influence of other tissues^17,18^. Our objective was to analyze specifically the impedance properties of skeletal muscle; since the epidermis and calcified scales on zebrafish would interfere with surface EIM of skeletal muscle, we used needle EIM.

In needle EIM, a low-amplitude, high-frequency alternating electrical current is applied through a pair of needle electrodes inserted into the muscle of interest, and the resulting voltages are measured via a second pair of needle electrodes, all in close proximity to one another. Myofiber or neuronal action potentials are not induced by the current^18,19^. Instead, compositional and structural features of bulk muscle tissue affect its electrical conduction properties, i.e. the muscle’s ability to resist or conduct electric current, and to store electric charge—termed bioelectrical impedance. Specific muscle features that impact bioelectrical impedance include muscle cell size (hypertrophy/atrophy), cell membrane integrity, fibrosis, edema, and fatty infiltration^20^. The relationship between applied current and recorded voltage in a muscle is used to determine its impedance characteristics, namely resistance (resistance to the current through intra- and extra-cellular ionic fluids), reactance (resistance due to the capacitance of cell membranes), and phase angle of the muscle (a geometric relationship between resistance and reactance that combines both features into a single measure that is less impacted by electrode distance or simple geometric alterations in the size of the tissue being measured)^20^. In human and animal models of muscle atrophy, EIM detects structural changes in diseased muscle including decreased cross-sectional myofiber area and increased space between myofibers, in addition to other parameters, which has been evidenced by a significant correlation between EIM values and morphometric endpoints^21–24^. However, in comparison to histological processing and quantitative software-assisted morphometric analyses of images of tissue sections which is not a trivial endeavor and requires weeks to complete, EIM measurements are captured in < 1 min and data analyses are completed in < 1 day.

EIM has been successfully employed in several species besides humans, including dogs^25,26^, rats^27,28^, and mice^29,30^. However, to our knowledge, the application of EIM to the assessment of zebrafish has not been reported. Zebrafish as a model organism is taking on increasing importance in the field of aging^31–35^. The zebrafish model has a number of advantages that make it an outstanding model to study age-related muscular disorders, including that females produce hundreds of offspring weekly and thousands of adult animals can be inexpensively housed in a modest footprint. Therefore, zebrafish are far more amenable to high-throughput chemical and genetic screening than rodent models. Moreover, due to their genetic and physiological similarities to humans, zebrafish are a well-established vertebrate organism to study human diseases^36–38^. Indeed, there is an especially high degree of conservation between humans and zebrafish in molecular and physiological pathways that regulate skeletal muscle biology^39–44^. Therefore, establishing the impedance characteristics of skeletal muscle in a zebrafish model of age-related atrophy will open a new experimentally tractable path of investigation in age-related sarcopenia.

Here, we evaluate the potential value of EIM as a tool to detect age-related sarcopenia in zebrafish. In young and aged zebrafish, we collected EIM data from the trunk musculature. In addition, established metrics of muscle function (swimming performance) and muscle quality (histological morphometrics) were quantified. As we show below, in aged animals, these cross-sectional analyses revealed that EIM parameters exhibited strong correlations with morphometric parameters of muscle atrophy, in addition to weak, short-duration movements during swimming. In sum, applying EIM to zebrafish skeletal muscle provides a fast (measurements captured in < 1 min), new approach to evaluating skeletal muscle health in a vertebrate animal model that is ideal for high-throughput disease assessment and therapeutic studies.

## Methods

### Ethical approval and ethics statement

All zebrafish studies and methods were performed in accordance with approved animal protocols from the Institutional Animal Care and Use Committees (IACUCs) at Beth Israel Deaconess Medical Center and Boston Children’s Hospital, in addition all studies and methods were performed in accordance with the Guide for the Care and Use of Laboratory Animals (National Research Council), the provisions of the Animal Welfare Act (USDA), and the National Institute of Health (NIH)/Public Health Service (PHS) Policy on Humane Care and Use of Laboratory Animals. This study was conducted as recommended by the ARRIVE guidelines. All procedures and analyses were randomized and blinded. The person performing the randomization was different from personnel conducting the experiments and analyses. However, it should be noted that aged animals trend toward increased weight as compared to young animals, with aged animals weighing 269 ± 126 mg more than young animals. As such, we cannot assume that the experimenter was completely blinded to the experimental group while collecting the EIM data and recording the videos. However, the experimenter was blinded to the animal’s experimental group during the analyses. Moreover, we did not attempt to blind the person conducting the experiments as to whether the fish were *casper* or *Tübingen* zebrafish since our interest was in determining the impact of aging on each strain and not on attempting to differentiate the strains using EIM.

### Zebrafish

Animals were maintained at 28.5 °C on a 14/10 h light/dark cycle, according to standard fish husbandry protocols. Adult *casper* and *Tübingen* zebrafish, wildtype strains, were each housed in mixed-sex tanks on a recirculating system. Aged *casper* fish were 33 months of age and young *casper* fish were 6 months of age. Aged *Tübingen* fish were 24 months of age and young *Tübingen* fish were 4 months of age. Approximately equal numbers of males and females were used.

### Morphometric measurements

Zebrafish body length was measured as the standard length (excluding the length of the caudal fin) using ImageJ. Body weight was measured by placing the animal on paper towels to remove excess water and weighing the animal on a tared scale. Kyphosis angle was measured in ImageJ. At the highest dorsal point of the outward curvature of the spine, a line was drawn that was parallel to the standard-length line. Next, the angle between this line and the dorsal aspect of the caudal muscular was measured in ImageJ (Supplementary Fig. 1).

### Swimming performance

Males and females were separated prior to the assay day. All fish were not fed prior to the assay to avoid possible effects of satiety. Testing occurred between 9 A.M. and 5 P.M. Fish were transported from the animal room to the procedure room and provided three hours acclimation time prior to the start of the assay. The testing room was 28 °C. The testing arena was 21 × 10 × 11 cm (L × W × H) with water filled to a height of 6 cm. All assay tanks were sandpapered to reduce reflection and black paper was placed at the bottom of the tank to increase the contrast between the transparent fish and the background (or white paper in the case of pigmented fish). The camera was positioned directly above the tank. Each zebrafish was gently netted and placed in the tank. Prior to recording videos, animals were given 10 min to acclimatize to the arena. The person performing the randomization was different from the person conducting the experiments so that the experimenter was partially blinded (due to the morphological differences between young and aged animals we cannot assume that the experimenter was completely blinded). Randomization was accomplished using a block randomization procedure, first assigning animals to blocks based on sex, and then randomly assigning each block (age). For each animal, digital video was captured for 3 min (30 frames/s) and subsequently analyzed using animal tracking software.

### Motion tracking and measurement of motor traits

EthoVision XT 17 (Noldus, Leesburg, VA) was used to track zebrafish motion and quantify motor traits: total distance traveled velocity, acceleration, turn angle, angular velocity, and lateral motion (see Supplementary Material). Detection settings that identify the object (i.e. animal) from the background, without subject loss due to misdetection (i.e. light reflections and shadows, etc.), were established by viewing the tracking performance accuracy in real-time. Track smoothing parameters were set to a maximum distance that would be improbable for a fish to move to in the time span of 1 frame (i.e. 30 ms); this prevents reflected light on the water or missing frames from confounding the measurement. For each frame, EthoVision extracted the (x,y) coordinates of the object’s geometric center in addition to the whole object’s surface area. The frame-by-frame raw data was exported for analysis. See Supplementary Material for additional details.

### Electrical impedance myography measurements

Zebrafish were euthanized in Tricaine S 500 mg/L. The EIM Needle electrode was made by inserting four 29 g Surepoint subdermal needles—conical variant (NDSDSURE, Lifesync Neuro, Coral Springs, FL) into four consecutive holes of a truncated 10-position Conn header with 1 mm spacing (S9214-ND, Digi-Key Electronics, Thief River Falls, MN) after the pins had been removed. The tips of the needles were inserted such that 1 mm protruded beyond the Header and their position was stabilized by embedding and baking the device in polymer oven-bake clay (Sculpey III), for 15 min at 130 °C. The needle array was then connected to an impedance measuring device (mView, Myolex, Inc, Boston, MA). The outer pair of electrode needles delivered alternating electrical current (400 µA, 1 kHz–1 MHz) while the inner pair measured the resulting voltage. The needle electrode was inserted into the epaxial caudal skeletal muscle below the dorsal fin (morphological landmark) (Supplementary Fig. 2). The puncture depth of the needle electrode is 1 mm, thus it can puncture the scales and epidermis, and enter into the skeletal muscle. Importantly, the electrode was positioned on the anteroposterior axis, in addition it was placed dorsal to major vessels on the anteroposterior axis so as not to puncture the dorsal aorta. When the needle was inserted, 4 serial measurements were captured within 15 s without moving the needle; measurement 1 and measurement 2 were compared to determine reproducibility. In a second analysis of reproducibility, these 4 measurements were then averaged to provide our first measurement value, the needle was then entirely withdrawn and then reinserted in the same location (as best as was possible) and 4 additional measurements taken; these were again averaged to provide our second measurement value (Supplementary Results and Supplementary Fig. 4M).

### Histological analysis of skeletal muscle

Zebrafish were fixed in Dietrich’s fixative for 72 h. Samples were paraffin-embedded and 10 µm sections were stained with hematoxylin and eosin. Cross-sectional images of the caudal trunk skeletal muscle were captured at 10× and 60× magnification on a Keyence BZ-X710 Imaging Platform. Using Keyence BZ-X Analyzer Software, the cross-sectional area of myofibers were measured using the hybrid cell counting tool. Automated segmentation was manually curated for accuracy. Incorrectly segmented features were manually adjusted using the fine edit tool. Six images of epaxial caudal muscle per animal were analyzed, at a site distal to the position of the needle electrode to avoid artifactual effects of needle injury. In zebrafish, slow-twitch (red) fibers are located in a small wedge-shaped band that runs parallel to the rostral-caudal axis of the body, while the fast-twitch (white) fibers are the majority of the muscle fibers in zebrafish skeletal muscle and are mainly arranged parallel to the body axis (Fig. 2C). The number of white fibers analyzed per animal was 600–1800 per animal.

### Impedance data parameterization

Multifrequency resistance and reactance data were fitted in MATLAB (The Mathworks, Natick, MA) to the Cole model^45^ of the frequency dependence of the impedance with the weighted complex nonlinear least squares method^46^ to yield the four standard Cole impedance parameters: Resistance at 0 and infinite frequency (R_0_ and R_∞_, respectively), the center frequency (f_c_) and alpha (α). These parameters offer information regarding alterations in muscle histology^19,23,47^. Separate models were used for low and high frequency components as this approach has been completed previously when assessing skeletal muscle given the two compositionally distinct types of myofibers (type 1 and type 2)^48^.

### Statistics

Data were analyzed in GraphPad Prism 9. Data are presented as mean ± standard deviation or mean ± standard error of the mean as marked. Motoric measurements, skeletal muscle morphometric measurements, and impedance data were all analyzed using Mann–Whitney tests. To adjust for multiple hypothesis testing, we applied a two-stage step-up method (Benjamini, Krieger and Yekutieli) for false discovery rate (FDR) adjustment with a threshold for statistical significance of q < 0.05. Correlation analysis was performed using Spearman tests. Reproducibility was assessed by measuring the mean percent difference between measurement 1 and measurement 2 (100 × (absolute value of measurement 1 − measurement 2)/mean of two measurements); the needle electrode was inserted and 4 serial measurements were acquired without moving the electrode, next measurement 1 and 2 were used to calculate mean percent difference. We note that we chose to use mean percent difference for assessing reproducibility over the often-used intraclass correlation coefficient (ICC) since the latter relies heavily on their being a wide range of values in the data (e.g., it is frequently used in disease conditions in which the animals/humans have a wide range of values based on varying disease severity). Here we were dealing with a relatively narrow dynamic range given that all the animals were nominally healthy; accordingly, the ICC would have provided a falsely low value.

## Results

### Morphometric characteristics

**Figure 1:**
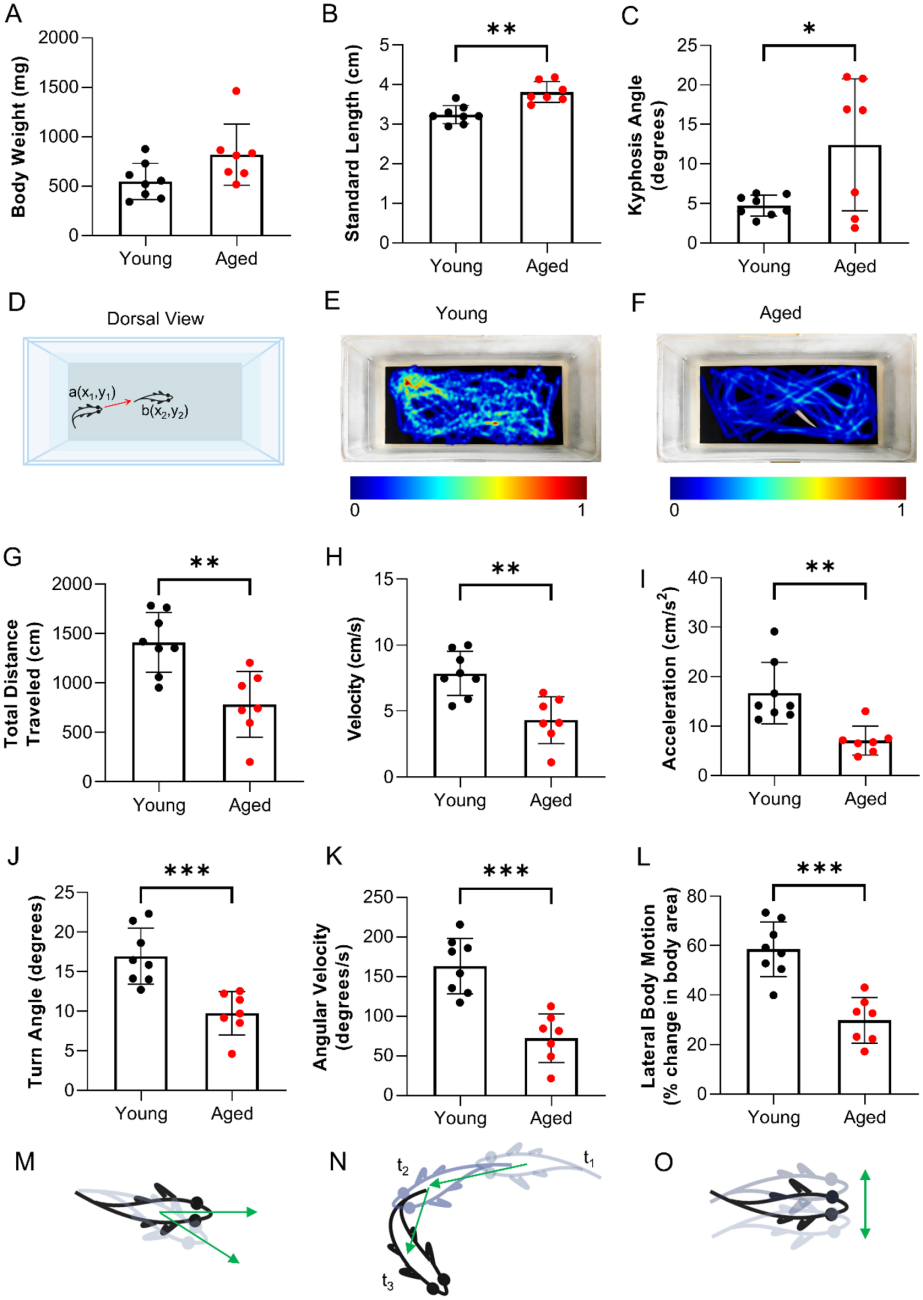
Swimming performance in adult zebrafish declines with age. Measurements of (**A**) body weight, (**B**) standard length, and (**C**) kyphosis angle in young and aged zebrafish. (**D**) Diagram of the testing area. (**E**,**F**) Heat maps depicting the time spent by animals in each (x,y) coordinate position. Locomotor measurements of (**G**) total distance traveled (q = 0.0039), (**H**) velocity (q = 0.0027), (**I**) acceleration (q = 0.0027), (**J**) turn angle (q = 0.0009), (**K**) absolute angular velocity (q = 0.0009), and (**L**) lateral body motion (q = 0.0013) (n = 7–8). Depiction of (**M**) turn angle, (**N**) angular velocity, and (**O**) lateral body motion. Data are presented as the mean ± SD.

The mean wildtype zebrafish lifespan is approximately 3.5 years, although some animals can live as long as 5^49^. Therefore, we chose 2–3 years of age to classify animals as “aged”. As for young animals, zebrafish become sexually mature adults at the age of 3 months. Therefore, we classified “young” animals as 4–6 months of age or 12.5% of their lifespan. Both male and female animals were used. As expected, aged fish (33 months of age) were larger than young fish (6 months of age). Aged zebrafish trended towards increased weight (820 ± 309 versus 550 ± 182 mg; p = 0.0721) and were longer (3.8 ± 0.3 versus 3.2 ± 0.2 cm, p = 0.0007) as compared to young zebrafish (Fig. 1A,B). Kyphosis, abnormal curvature of the spine, increases with age and is quantified using kyphosis angle. All young zebrafish exhibited a normal curvature of the spine while aged zebrafish exhibited an increase in mean kyphosis angle (4.7 ± 1.3 versus 12.4 ± 8.3°, p = 0.05 Fig. 1C).

### Swimming performance in adult zebrafish declines with age

Zebrafish exhibit a robust repertoire of stereotypical motor traits^50^. Several parameters of swimming performance have been successfully used to assess muscle function in zebrafish models of muscular dystrophies^51–53^. To evaluate the impact of aging on swimming performance, we captured digital videos of zebrafish and, using animal tracking software, measured parameters including total distance traveled, velocity, and turn angle (Fig. 1D–O). Aged zebrafish exhibited decreased total distance traveled (784 ± 332 versus 1409 ± 302 cm; p = 0.003), decreased velocity (6.50 ± 3.08 versus 11.99 ± 3.92 cm/s; p = 0.002), and decreased acceleration (7.07 ± 2.92 versus 16.67 ± 6.23 cm/s^2^; p = 0.002) as compared to young zebrafish (Fig. 1G–I; all FDR ≤ 0.05). Young zebrafish were capable of body maneuvers that resulted in deeper turn angles as compared to aged fish (16.94 ± 3.54 versus 9.74 ± 2.74°, p = 0.0003, FDR ≤ 0.05; Fig. 1J,M). In addition, young fish exhibited greater angular velocity as compared to aged fish, indicating that it takes more time for aged fish to change their direction of travel (163.52 ± 35.08 versus 72.44 ± 30.75°/s, p = 0.0003, FDR ≤ 0.05; Fig. 1K,N). Lastly, aged zebrafish exhibited decreased lateral motion, i.e. non-displacement motion, as compared to young zebrafish (29.81 ± 9.22 versus 58.57 ± 11.10% change in body area, p = 0.0006, FDR ≤ 0.05; Fig. 1L,O). This indicates that aged fish exhibit reduced “in place” body contortions. In sum, these findings demonstrate that aged zebrafish exhibit defects in stereotypic motor traits as compared to young zebrafish which result from weak, short-duration movements in aged fish as compared to fast, vigorous movements in young fish.

### Age-related atrophy of skeletal muscle myofibers in zebrafish

**Figure 2:**
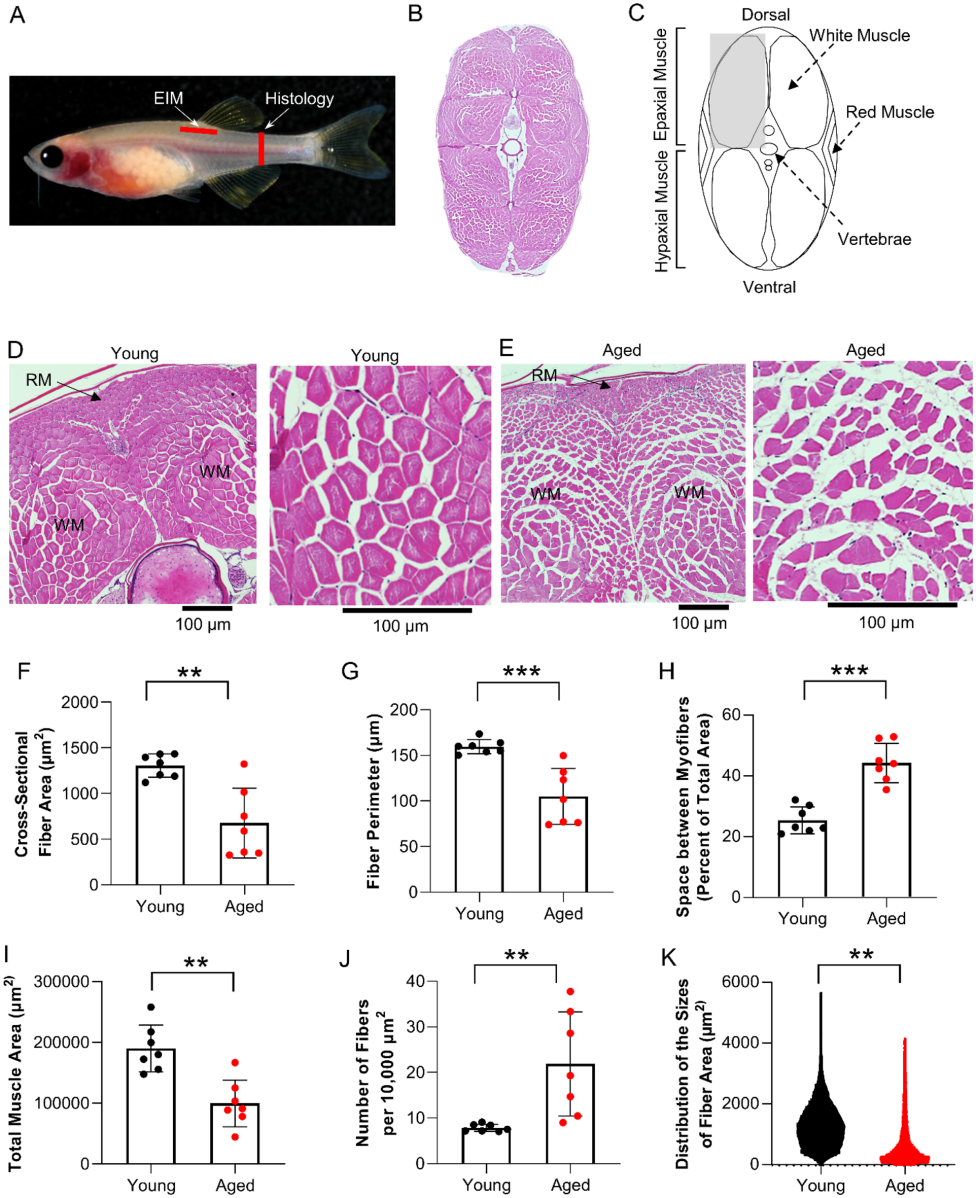
Age-related atrophy of skeletal muscle myofibers in zebrafish. (**A**) Red bar denotes the cross-sectional position for histological analysis of caudal skeletal muscle tissue, and (**B**) a representative H&E-stained section of that region. (**C**) Diagram of caudal musculature in zebrafish including the location of epaxial and hypaxial muscles. The gray box denotes the area used for histological analysis. Representative H&E images from (**D**) young and (**E**) aged zebrafish. Muscle fiber morphometric measurements of (**F**) cross-sectional fiber area (q = 0.0043), (**G**) fiber perimeter (q = 0.0015), (**H**) space between myofibers (q = 0.0015), (**I**) total muscle area (q = 0.0004), (**J**) number of fibers per unit area (q = 0.0020), and (**K**) distribution of the sizes of fiber area in young and aged animals (n = 7–8). Data are presented as mean ± SD.

To assess muscle architecture in aged zebrafish, we obtained H&E sections from the caudal musculature at a site distal to the location of the electrode array (Fig. 2A–C). Young zebrafish exhibited large polygonal-shaped muscle cells that were ensheathed in a layer of ordered endomysium (connective tissue), while aged zebrafish exhibited smaller muscle fibers with disordered endomysium and increased extracellular spacing between muscle fibers (Fig. 2D–E, Supplementary Fig. 3). We quantified these observations using image analysis software. Morphometric features of fibers were quantified in epaxial muscle (Fig. 2C). Mean cross-sectional fiber area and fiber perimeter were reduced in aged animals as compared to young animals (676 ± 383 versus 1304 ± 128 µm^2^, p = 0.004; and 104.98 ± 30.61 versus 159.64 ± 7.70 µm, p = 0.0005, respectively, FDR ≤ 0.05; Fig. 2F–G). Aged animals exhibited increased extracellular space between myofibers as compared to young animals (25.42 ± 4.44 versus 44.28 ± 6.44% of total area, p = 0.0005, FDR ≤ 0.05; Fig. 2H). Total muscle area was greater in young animals (190,225 ± 38,395 versus 99,767 ± 38,526 µm^2^, p = 0.002, FDR ≤ 0.05; Fig. 2I) but total fiber number per unit area was greater in aged animals (7.85 ± 0.81 versus 21.89 ± 11.43 fibers per 10,000 µm^2^, p = 0.001, FDR ≤ 0.05; Fig. 2J). The increased number of fibers per unit area was driven by the distribution of fiber size in aged versus young animals; aged animals exhibit a greater number of smaller fibers (Fig. 2K). Together, these morphometric features of muscle architecture demonstrate age-dependent muscle atrophy in zebrafish.

### Electrical impedance myography detects altered phase angle, reactance, and resistance in the caudal muscles of aged zebrafish

**Figure 3:**
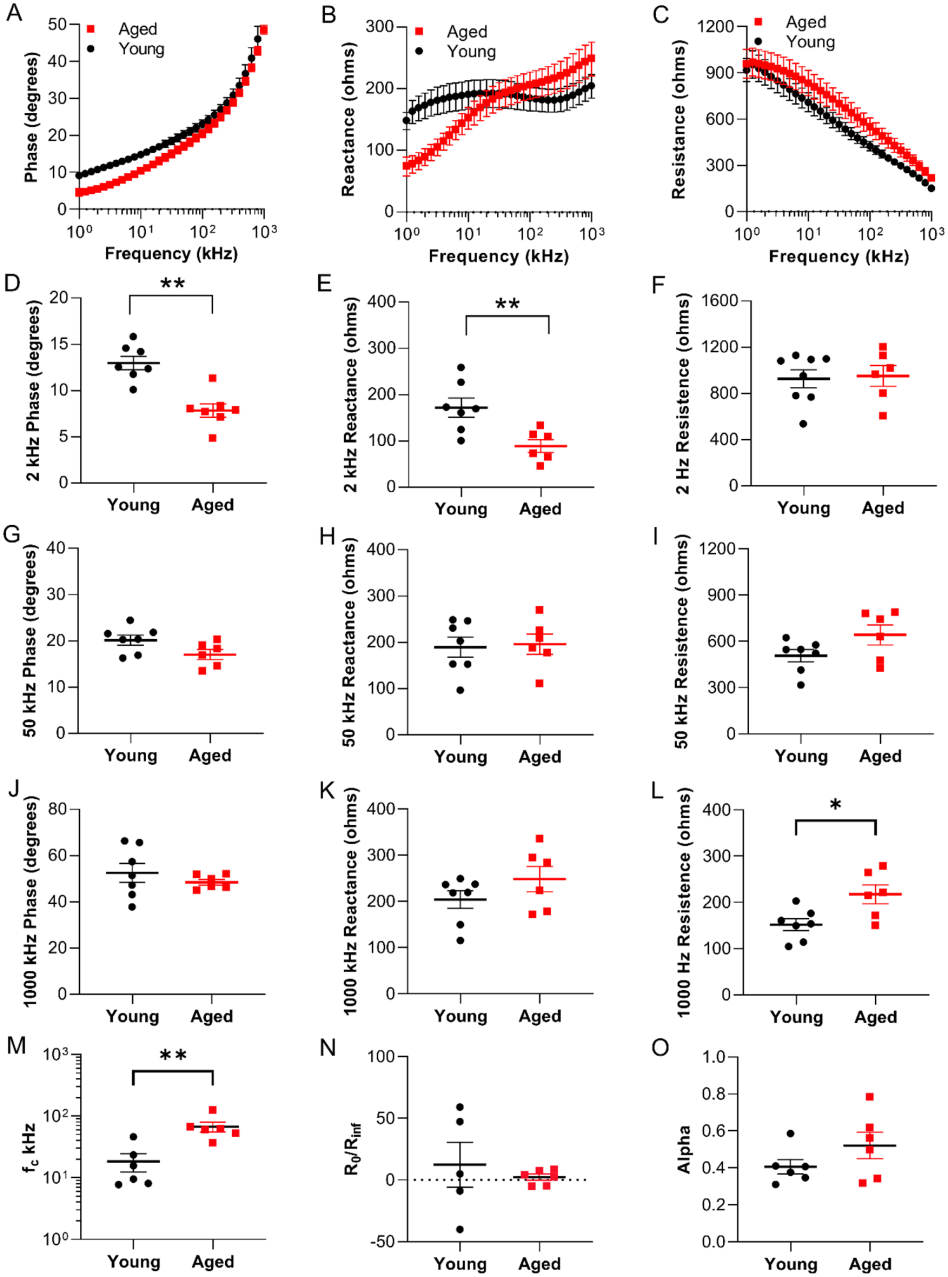
Electrical impedance myography detects age-related muscle changes in zebrafish. Multifrequency graphs (1 kHz–1 MHz) for (**A**) phase, (**B**) reactance and (**C**) resistance. Single frequency analyses at 2, 50 and 1000 kHz. (**D**) 2 kHz phase (q = 0.0085), (**E**) 2 kHz reactance (q = 0.0371), (**F**) 2 kHz resistance, (**G**) 50 kHz phase, (**H**) 50 kHz reactance, (**I**) 50 kHz resistance, (**J**) 1000 kHz phase, (**K**) 1000 kHz reactance , (**L**) 1000 kHz resistance (not FDR significant but nominally significant), (**M**) Cole parameter f_c_ (q = 0.0181), (**N**) Cole parameter ratio R_0_/R_∞_, and (**O**) Cole parameter alpha (n = 6–7). Data are presented as mean ± SEM.

EIM across a range of frequencies (1 kHz–1 MHz) was measured in the caudal muscles of young and aged zebrafish (Fig. 3A–C). In aged animals, there was an overall trend toward decreased phase and increased resistance as compared to young animals, whereas reactance values were lower in aged animals at frequencies below 60 kHz and then were higher at frequencies beyond that value. Single frequency 2 kHz analyses in aged animals showed significantly decreased phase angle (5.3 ± 2.1 versus 10.7 ± 1.5 kHz; p = 0.001, FDR ≤ 0.05) and reactance (89.0 ± 3.9 versus 172.2 ± 54.8 ohms; p = 0.007, FDR ≤ 0.05), while resistance was normal as compared to young animals (Fig. 3D–F). At 50 kHz, the most “standard” single frequency for EIM measurement, there were no significant differences, although phase was lower and resistance higher in the older animals (Fig. 3G–I). At 1000 kHz, aged animals exhibited nominally significantly increased resistance (217.5 ± 20.5 versus 151.6 ± 12.9 ohms; p = 0.035, not FDR significant), while phase angle and reactance did not change as compared to young animals (Fig. 3J–L). Next, we tested a second independent cohort of animals and impedance measurements in a second cohort of young and aged animals demonstrated similar findings (Supplementary Fig. 4A–L); we also tested a larger sample size (double the size) and found similar findings (Supplementary Fig. 7). Reproducibility was assessed at 2 kHz by determining the mean percentage difference between two consecutive measurements for each EIM parameter in the cohort. The mean percentage difference for phase, reactance and resistance was 5.34 ± 1.17%, 6.25 ± 1.12%, and 2.95 ± 0.43% respectively. Data for additional frequencies are shown in Supplementary Table 5. To further assess reproducibility, 4 tests were captured, the needle electrode was removed and then reinserted, and an additional 4 tests were captured (Supplementary Fig. 4M).

Next, multifrequency resistance and reactance data were fitted to the Cole model to calculate the four standard Cole impedance parameters. The Cole impedance parameters are modeled parameters^45^ that inform on alterations in muscle histology since resistance at 0 frequency (R_0_) and resistance at infinite frequency (R_∞_) are both associated with cell density, while center frequency (f_c_) is associated with myofiber size and alpha (α) with cell size distribution^19,23,47^. Based on impedance values < 100 kHz, there was a significantly higher f_c_ in aged *casper* animals (18.4 ± 6.1 versus 70.4 ± 14.8, p = 0.0087, FDR ≤ 0.05; Fig. 3M). A higher f_c_ generally is consistent with smaller cell size. Concordantly, histological analysis demonstrated smaller cell sizes in aged animals as compared to young animals (Fig. 2D–F). Alpha, resistance at 0 frequency and resistance at infinite frequency (α, R_0_ and R_∞_) were not different between groups (Fig. 3N,O, Supplementary Table 1). In sum, the EIM data indicate that 2 kHz phase, 2 kHz reactance, and the Cole parameter f_c_ detect changes indicative of atrophy in the skeletal muscle tissue of aged *casper* zebrafish. Cole parameters were also determined for the *casper* zebrafish using impedance values > 100 kHz (Supplementary Table 2). No significant differences were found in any of the high frequency Cole parameters when values for the young and aged *casper* zebrafish were compared. Both low (< 100 kHz) and high (> 100 kHz) frequency Cole parameters were also determined for the *Tübigen* zebrafish using impedance data found in Supplementary Fig. 7. Low and high frequency Cole parameters for the *Tübigen* zebrafish can be found in Supplementary Tables 3 and 4, respectively. Based on impedance values < 100 kHz, there was a significantly higher f_c_ in aged *Tübigen* animals (6.68 ± 1.2 versus 60.4 ± 18.9, p < 0.0001), in agreement with the trend reported for the *casper* zebrafish.

### Cross-sectional myofiber area in aged zebrafish correlates with electrical impedance myography measurements in aged animals

**Figure 4:**
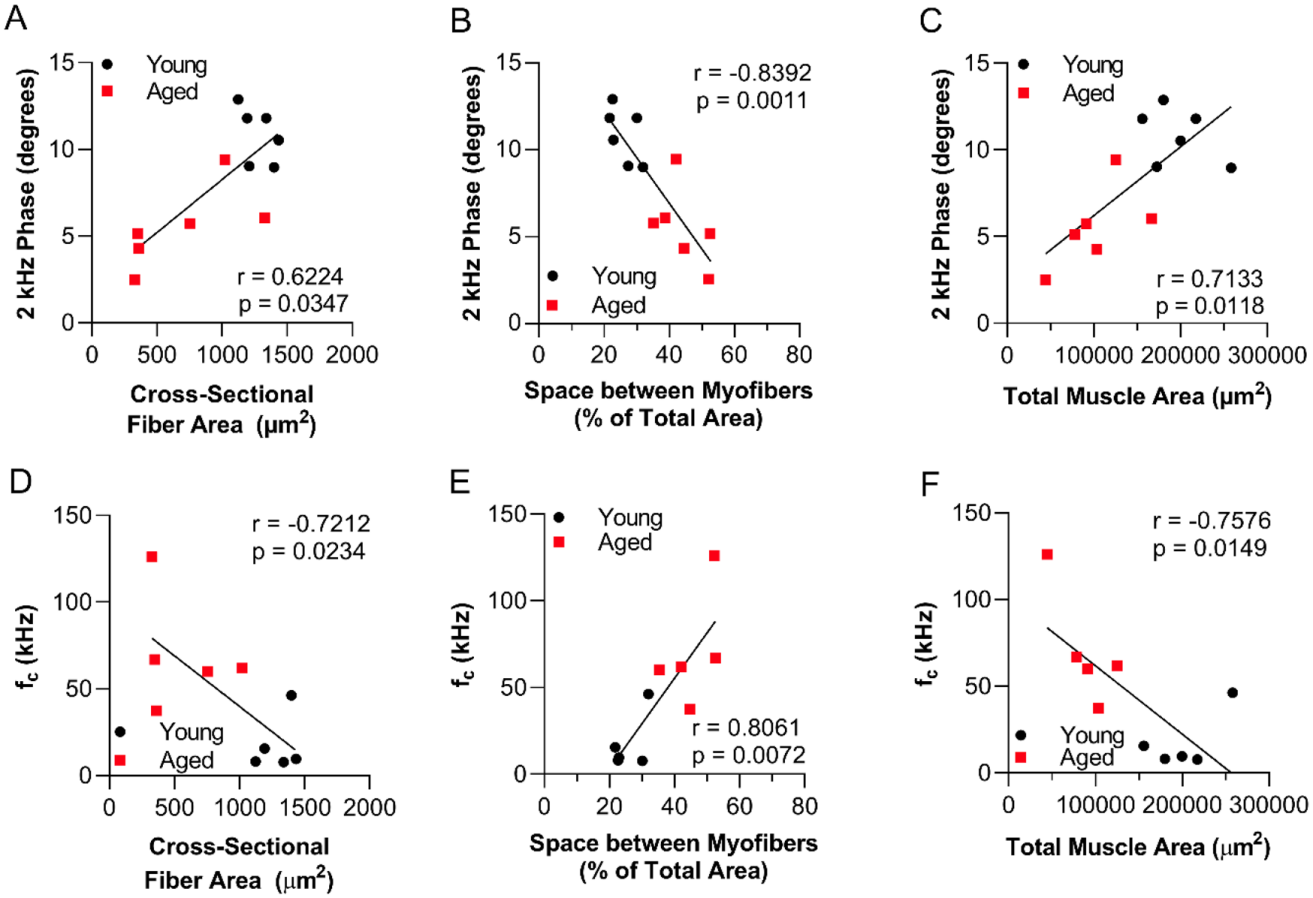
Age-related changes in zebrafish muscle morphology correlate with electrical impedance myography measurements in caudal muscle. Correlation between 2 kHz phase values and morphological features: (**A**) cross-sectional fiber area, (**B**) space between myofibers, and (**C**) total muscle area for young and aged zebrafish combined. Correlation between Cole parameter f_c_ and (**D**) cross-sectional myofiber area, (**E**) space between myofibers, and (**F**) total muscle area for young and aged zebrafish data combined. Spearman r and p values are shown in each panel.

In mammalian models, electrical impedance correlates with histological and morphometric features of muscle fibers, and are biomarkers of aging^21,22,54,55^. The 2 kHz phase values showed significant correlations with basic histological features including total muscle area (r = 0.7133, p = 0.011), space between fibers (r = − 0.8392, p = 0.0011), and cell size (r = 0.6224, p = 0.0347), with the strongest correlation with space between myofibers (Fig. 4A–C). Concordant with the correlation between 2 kHz phase and histological features of cell size, the Cole parameter *f*_*c*_, which is generally considered the most closely aligned to myofiber size, was also correlated with total muscle area (r = − 0.7576, p = 0.0149), space between fibers (r = 0.8061, p = 0.0072), and cell size (r = − 0.7212, p = 0.0234) (Fig. 4D–F).

### Age-related changes in swim performance correlate with electrical impedance myography measurements

**Figure 5:**
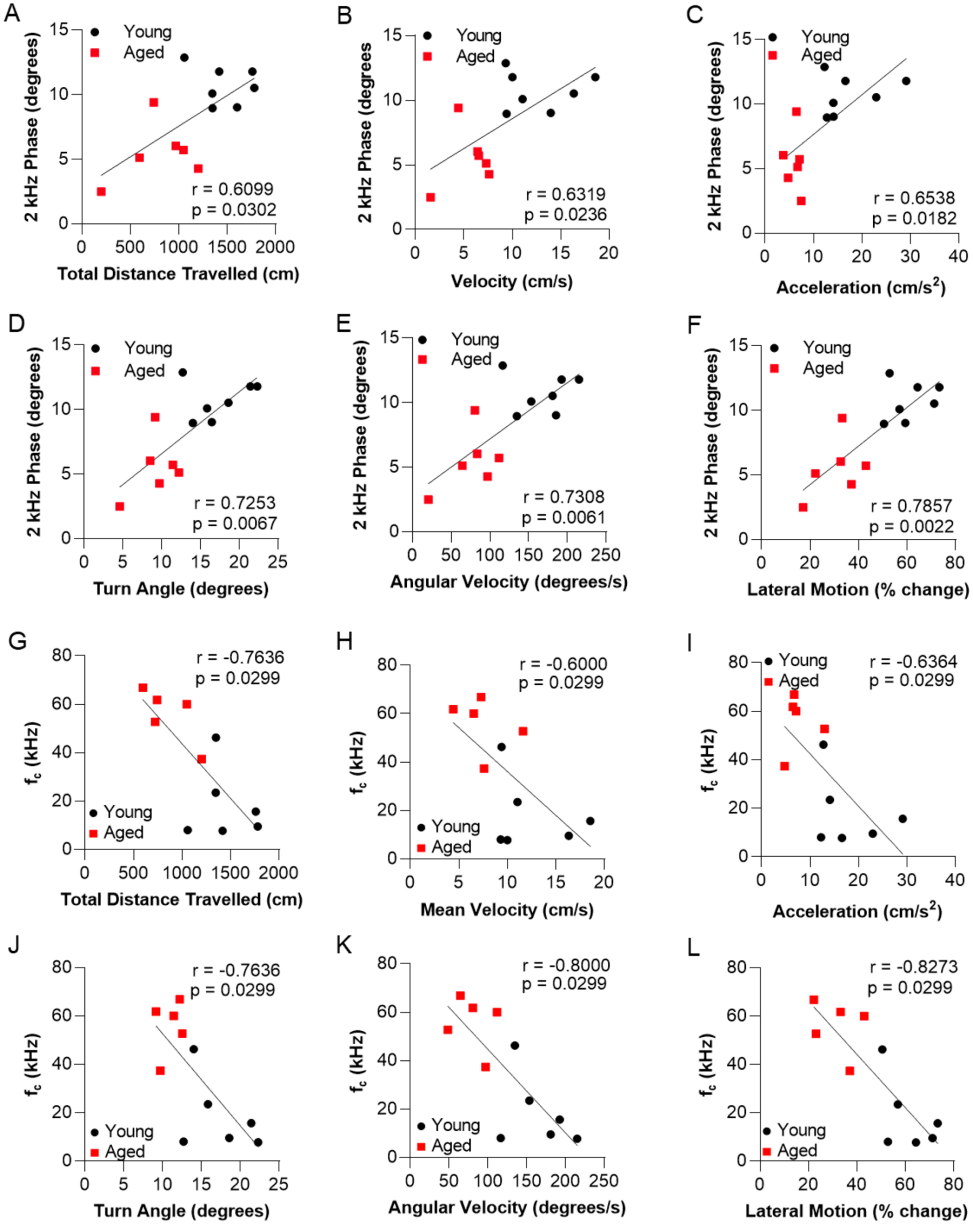
Swim performance deficits in aged zebrafish correlate with electrical impedance myography measurements of caudal muscle resistance. Correlation between 2 kHz phase values and motor traits: (**A**) total distance traveled, (**B**) velocity, (**C**) acceleration, (**D**) turn angle, (**E**) angular velocity, and (**F**) lateral motion for young and aged zebrafish data combined. Correlation between f_c_ (kHz) values and motor traits: (**G**) total distance traveled, (**H**) velocity, (**I**) acceleration, (**J**) turn angle, (**K**) angular velocity, and (**L**) lateral motion for young and aged zebrafish data combined. Spearman correlation r and p values are shown in each panel.

The biophysics of locomotor maneuvers in zebrafish has been described and requires the coordination of trunk musculature during swimming^56^. As with the histological features of muscle fibers, including both the old and young animals together, there were strong correlations between 2 kHz phase and every measurement of locomotor function (Fig. 5A–F). The strongest correlations with 2 kHz phase were for turn angle (r = 0.7253, p = 0.0067), angular velocity (r = 0.7308, p = 0.0061), and lateral motion (r = 0.7857, p = 0.0022; Fig. 5D–F). Additionally, a replication cohort of young and aged animals demonstrated similar correlations between motor traits and 2 kHz phase (Supplementary Fig. 5). There were also correlations, albeit moderate, with 2 kHz reactance and turn angle, angular velocity, and lateral motion (r = 0.5714, p = 0.0449; r = 0.5934, p = 0.036; r = 0.5879, p = 0.0381, respectively). At low frequencies, resistance was not correlated with any motor trait. By contrast, at high frequency, 1000 kHz resistance was inversely correlated with turn angle, angular velocity, and lateral motion (r = − 0.6044, p = 0.032; r = − 0.6538, p = 0.0182; r = − 0.7033, p = 0.009, respectively). The Cole parameter f_c_ was strongly correlated with angular velocity (r = − 0.800, p = 0.0047) and lateral motion (r = − 0.8273, p = 0.0027) (Fig. 5G–L). These findings demonstrate that, in addition to changes in histological features of muscle morphology, EIM measurements at 2 kHz phase and the Cole parameter f_c_ are biophysical metrics that detect defects in motor traits and swimming performance between aged and young zebrafish. Finally, swimming performance is also correlated with cross-sectional fiber area (Supplementary Fig. 6).

## Discussion

Zebrafish are diurnal, vertebrate animals that experience gradual, age-related decline in cognitive function, musculoskeletal stability, and neuroendocrine homeostasis. In this study, aged zebrafish exhibited reduced mobility, flexibility, and swim performance as evidenced by decreased velocity, turn angle/angular velocity, and acceleration. In addition to defects in locomotor functions, histological analyses of muscle fibers from aged and young animals showed morphological features of muscle atrophy including decreased myofiber size and increased extracellular space between fibers. Aged and young muscle tissue also exhibited divergent bioelectrical impedance properties. At low frequencies (2 kHz), quantitative measures of motoric traits correlated strongly with lower phase angle. Moreover, there was a strong correlation between established metrics of muscle function (swimming performance) and muscle quality (histological features) with 2 kHz phase values. Additionally, several histological features and motor traits were also moderately correlated with reduced reactance values. Importantly, aged zebrafish recapitulated both the morphological and bioelectrical features of muscle degeneration observed in mice and humans^21,30,31^. Together, these findings demonstrate that EIM in zebrafish is a fast, sensitive method for quantifying changes in organismal muscle function and quality.

Electrical impedance refers to the full properties of a tissue or substance to impede the flow of electrical current and represents a substantial area of long scientific inquiry (see^19^ for an extended overview of the subject). In contrast to a simple resistance measure, impedance also incorporates the capacitive properties of the tissue and thus impedance is a more informative measurement that includes both a real component (resistance, R) and an imaginary component (reactance, X). The overall impedance can be calculated via the equation Z = sqrt (X^2^ + R^2^) but it is generally not employed in most bioimpedance work since the resistive values, being relatively large, overwhelm the reactive components. Therefore, the phase angle (or simply phase), obtained from the trigonometric relationship phase = arctan (reactance/resistance), is more useful and can effectively combine the two measures. The phase calculation also helps reduce the impact of simple morphometric differences when comparing different sized or spaced electrodes or when different samples are being assessed. As electrical current can be passed over a wide range of frequencies, termed impedance spectroscopy, the frequency dependence of the observed impedance is also important as different tissues can exhibit characteristic values and peaks. For example, healthy skeletal muscle typically demonstrates a peak reactance in the 20–80 kHz range^20^, and, concordantly, we observed peak reactance in young zebrafish muscle at 20 kHz.

Although several factors impact the nature of the electrical properties, including the geometry of the electrode array itself and the tissues being measured, in addition to other factors, some general rules can be applied to the interpretation of impedance data. Lower frequencies (e.g. under 20 kHz) generally interrogate only the extracellular compartment whereas higher frequencies (e.g. 200 kHz or higher) will interrogate the entire tissue volume (both extra and intracellular components fully). Intermediate frequencies (20–200 kHz) reflect increasing intracellular contributions as the frequency increases. The majority of the results in zebrafish are generally consistent with impedance theory. The reduced reactance and phase at 2 kHz in aged zebrafish are consistent with reduced cell size and increased space between fibers, which was confirmed by histological analysis. These findings are also concordant with known aging related changes in muscle morphology in mice, and also the reduced low frequency reactance and phase observed in aged mice^30^. Moreover, deficits in stereotypic motor traits, used to assess swimming performance in zebrafish, were also strongly associated with 2 kHz phase. These findings are concordant with murine studies demonstrating that in older mice, a reduction in muscle size contributed to altered functional behaviors, which, importantly, was detected by reduced low frequency phase^30^. It is also known that increased space within the muscle deeply impacts the impedance data, since it provides a low capacitive path through the muscle, thus greatly reducing reactance and phase at low frequencies. Aged zebrafish exhibited significantly increased space between muscle fibers as compared to young animals, as evidenced by histological analysis, which likely contributes to the reduction in reactance and phase in aged zebrafish. Finally, the increased resistance at high frequencies in aged zebrafish is also consistent with deposition of a less conductive material throughout the muscle.

Impedance values also reflect architectural features of tissues. Cell membranes more substantially impact reactance whereas resistance tends to be most sensitive to any histological feature that reduces the conductivity of the tissue (e.g. fat and connective tissue). The Cole impedance model was developed to help interpret the impedance behaviors by fitting the spectroscopic data to a model, with specific outcomes including a center frequency (assessing cell size), alpha (providing information on the distribution of cells), and resistance at 0 and infinite frequencies (providing a measure of pure extracellular resistance and the entire mass resistance, respectively). And, indeed, we found a strong relationship between the center frequency and morphological assessment of the muscle. We did not expect strong variation in myofiber size in either animal group, and thus it is unsurprising that alpha was not different between the groups. The lack of a reduced resistance at 0 frequency in the older fish is unexpected, given the increased space between fibers. However, that parameter could be offset by the space being filled with relatively poorly conductive material.

Our protocol was reproducible, both within a subject and between independent cohorts. In serial tests on the same animal, we observed a 3–6% variability in impedance measurements. Moreover, impedance measurements in a second cohort of young and aged animals demonstrated similar findings; aged animals exhibited decreased phase and reactance at low frequencies. There were some interesting differences in the impedance data at high frequencies between the two cohorts. In murine models, this pattern has been associated with increased fat content^55,57^. In our study, the fixation and embedding process extracted lipids, therefore future studies will be required to compare adipose tissue and lipid content between these two strains. However, we can speculate that this may be due to genetic differences in intermuscular adipose tissue or intramyocellular lipids between these strains, although to our knowledge this has not been reported. Alternatively, these findings may be due to nutritional differences in the husbandry of the two wildtype strains. *casper* fish (Boston Children’s Hospital Zebrafish Facility) were fed Techiplast Zebrafeed pellet food (10% of their body weight, once per day) while *Tübingen* fish (Beth Israel Deaconess Medical Center Zebrafish Facility) were fed live Artemia (5% of their body weight, twice per day). Future Oil Red O studies and triglyceride measurements are required to determine if there is a difference in fat content between *casper* fish and *Tübingen* fish.

In perspective, our findings are very consistent with reported literature on EIM for mice. EIM measurements in young versus aged mice demonstrated similar trends in EIM and muscle function as in the zebrafish model^30^. Similar to aged zebrafish, the gastrocnemius muscle of aged mice exhibited lower impedance phase and reactance values. In addition, similar to aged zebrafish, EIM parameters in aged mice correlated with established metrics of muscle function^30^. While rodent models have demonstrated the potential of EIM to detect subtle disease related phenotypes in muscle health, there are unique features of the zebrafish model that make it a powerful tool for studying muscle biology. Moreover, there are several advantages to zebrafish as compared to mice and rats. One advantage of the zebrafish over rodents is that zebrafish are diurnal. Thus, zebrafish have a more similar circadian rhythm to humans than rodents. Second, an interesting anatomical difference between zebrafish and mice/humans, is that in zebrafish slow- and fast-twitch fibers are not intermixed in skeletal muscle. Due to anatomical compartmentalization of slow- and fast-twitch fibers in zebrafish (Fig. 2C), specialized antibodies are not required to identify slow- and fast-twitch fibers while in mice and humans immunohistochemical staining with anti-myosin heavy chain type I and anti-myosin heavy chain type II antibodies are required. Moreover, age-related atrophy of skeletal muscle in humans is primarily due to changes in fast-twitch fibers as opposed to slow-twitch fibers^58,59^; thus the anatomy of zebrafish enables a straightforward and unambiguous approach to focusing on fast-twitch fibers. Second, the zebrafish model offers an increased and more efficiently scalable platform as compared to mice, producing hundreds of embryos every week in a small footprint; thus, chemical screens using libraries of > 10,000 small molecule are feasible. Third, leveraging recent advances in genome editing in zebrafish, hundreds of disease-related genes can be evaluated^60–62^. Thus, zebrafish are uniquely positioned for high-throughput phenotypic-based screening and the discovery of novel lead compounds to advance to clinical trials^63^. Concomitantly, in the last decade, there has been a rise in phenotypic-based drug discovery in academic and pharmaceutical environments. This is because twice as many first-in-class molecular entities that received FDA approval were advanced from phenotypic-based screens, as opposed to the traditional target-based approaches^64^. Importantly, in our study, we demonstrated that a sample size of 8 is powered to detect an effect between experimental groups in zebrafish (Supplementary Figs. 4 versus 7). This number is important because there is a “sweet spot” of phenotypic richness and scalability in zebrafish chemical screening. The sample size, established in this study, combined with the speed at which EIM measurements can be captured (< 1 min) will enable the testing of numerous animals per day. In sum, our study provides the foundation for future chemical screens in zebrafish using impedance data as the endpoint to assess muscle health. Moreover, by combining a fast (measurements captured in less than 1 min), precise, and data rich endpoint, with the advantages of zebrafish, we have opened a new path to accelerate the discovery of small molecule therapeutics for sarcopenia, as well as potentially other neuromuscular disorders.

In future work, we also plan to develop a surface EIM method in zebrafish. While needle EIM allowed us to acquire electrical impedance data from skeletal muscle without the influence of other tissues, the needle method is not amenable to longitudinal assessments in very small animals. However, EIM can be applied using noninvasive approaches i.e. surface EIM; using this method it also becomes possible to follow subjects longitudinally over time to monitor disease progression or evaluate response to therapy^23,24^. To accomplish this in zebrafish, we ideally will need to conduct impedance studies of the biophysical (i.e., resistive and capacitive) properties of the epidermis and scales themselves and their contribution to the overall measured signal.

This study has a number of important limitations. First, our study only included two age groups. Therefore, we do not know the precise age when the muscular defects develop nor the longitudinal trajectory in the development of gradual muscle changes through lifespan. Second, we used the minimum number of animals required to reproducibly detect differences between groups, guided by our prior EIM studies in mice in which we have used similar sample sizes^65–67^. We also used two complementary assays to validate our EIM findings: histology and swimming performance. In addition, we replicated our EIM findings in an independent cohort (Supplementary Material). However, it is possible that an increased sample size would detect additional subtler differences between groups; accordingly, to assess this, we doubled our sample size (Supplementary Figs. 4 and 7)  but found that doubling sample size did not detect additional EIM differences. Third, as skeletal muscle is highly structured, its anisotropy is very strong^20^. However, we did not attempt to capture that in this early work. It is likely the data gathered here represent some value intermediate to a true longitudinal measurement (current flowing parallel to the muscle fibers) and a true transverse measurement (current flowing perpendicular to the muscle fibers), given the orientation of fibers in the trunk region measured relative to placement of the electrode array. Fourth, we conducted studies in the caudal muscle; therefore, future studies are required to determine if there are muscle-region specific effects of aging in zebrafish.

Establishing EIM in zebrafish in combination with identifying the bioelectrical abnormalities in a model of age-related sarcopenia, provides new opportunities to evaluate potential treatment approaches for muscle disorders. Our future studies include chemical screens in sarcopenic zebrafish, in addition to disease-specific genetic models that impact muscle health, including primary neuromuscular diseases. Specifically, we plan to leverage our platform to study amyotrophic lateral sclerosis using established genetic models in zebrafish^68^. In addition, the development of a surface probe and measurement techniques for zebrafish are currently in progress. This next-generation methodology will provide an opportunity to conduct repeated, spatiotemporal measurements on the same animal through lifespan and monitor the trajectory of disease development or therapy effect.

### Impact statement

Establishing the application of electrical impedance myography to the zebrafish model merges the advantages of a fast, sensitive, high-content tool with the experimental tractability of zebrafish. This will enable chemical screening of candidate therapeutics for sarcopenia, for which there is currently no treatment. Finally, the bioelectrical properties identified in aged zebrafish may also serve as a biomarker for studies aimed at interrogating the pathomechanisms of neuromuscular disorders.

## Supplementary Information

Supplementary Information.

## Data Availability

The datasets generated in the current study are available from the corresponding author on reasonable request.
