# Supplementary material for: Electrical impedance myography detects age-related skeletal muscle atrophy in adult zebrafish
Source: Sci Rep. 2023 May 3;13:7191. doi: 10.1038/s41598-023-34119-6 (PMC10156759; doi:10.1038/s41598-023-34119-6)
Supplement: Supplementary file 1 — Supplementary Information. [file 41598_2023_34119_MOESM1_ESM.pdf]

SUPPLEMENTARY MATERIAL

Electrical Impedance Myography Detects Age-Related Skeletal Muscle Atrophy in Adult Zebrafish

Seward B. Rutkove[1,2,‡], Santiago Callegari[3], Holly Concepcion[1], Tyler Mourey[4], Jeffrey Widrick[2,5], Janice A. Nagy[1], and Anjali K. Nath[2,3,6,‡]

[1]Department of Neurology, Beth Israel Deaconess Medical Center, Boston, MA, 02115, USA.
[2]Harvard Medical School, Boston, MA 02115, USA.

[3]Department of Cardiology, Beth Israel Deaconess Medical Center, Boston, MA 02115, USA.
[4]Zebrafish Core Facility, Beth Israel Deaconess Medical Center, Boston, MA 02115, USA.
[5]Division of Genetics and Genomics, Boston Children's Hospital, Boston, MA 02115, USA.
[6]Broad Institute, Cambridge, MA 02142, USA.
[‡]To whom correspondence should be addressed: Anjali K. Nath (anath1@bidmc.harvard.edu) and Steward B. Rutkove (srutkove@bidmc.harvard.edu)

SUPPLEMENTARY METHODS

Calculating kyphosis angle. Kyphosis angle was measured in ImageJ by using morphological landmarks to create guidelines. At the highest dorsal point of the outward curvature of the spine, a line was drawn that was parallel to the standard-length line (**Supplementary Figure 1**, green lines). Next, a line was drawn at the anterior side of the operculum (**Supplementary Figure 1**, outlined with a white dotted line). The angle between the green line and the dorsal aspect of the caudal musculature was measured (**Supplementary Figure 1**, yellow).

Electrical impedance myography in zebrafish: Experimental set-up. Zebrafish were placed under a magnifying lens. The needle electrode has four 29-gauge pins that are spaced 1 mm apart (total probe width = 3 mm). The electrode was placed in the caudal musculature just below the dorsal fin – a morphological landmark that was used to consistently insert the electrode in the same region between animals (**Supplementary Figure 2A**). A low-amplitude, high-frequency alternating electrical current is applied to the muscle through the pair of outer electrodes, and the resulting voltages are measured via a second pair of inner electrodes (**Supplementary Figure 2B**). The procedure was completed in <1 minute.

Analysis of EIM reproducibility. Reproducibility was assessed by measuring the mean percent difference between measurement 1 and measurement 2 (100 x (absolute value of measurement 1 - measurement 2)/mean of two measurements). We inserted the needle electrode, and with the needle electrode in place, we did 4 serial measurements without moving the electrode (measurement 1 = the mean of the 4 serial tests). We then removed the needle electrode entirely and then reinserted it and did 4 additional serial measurements (measurement 2 = the mean of the 4 serial tests). Reproducibility was also measured within each needle electrode insertion (i.e. the 4 serial tests taken without moving the electrode).

Behavioral data acquisition (EthoVision). Movement variables (distance, velocity, and acceleration) are based on the change in X and Y coordinates between two or more consecutive frames. The direction variables (turn angle and angular velocity) are based on the change in the heading of the center point of the animal across three frames. The body variable (mobility) is based on the differential change in area (pixels) of the animal between consecutive frames.

Distance is defined as the distance traveled by the center of the body, frame-by-frame. The total distance traveled is the cumulative sum of all the values and was calculated with the following equation:

$$DM_n = \sqrt{(X_n - X_{n-1})^2 + (Y_n - Y_{n-1})^2}$$

Where DM_n is equal to the distance moved by the center of the body between frame n–1 and frame n. X_{n-1} and Y_{n-1} are the X and Y coordinates of the center point at frame n–1. X_n and Y_n are the X and Y coordinates of the center point at frame n.

Velocity refers to the distance traveled by the center of the body per unit of time, and was calculated using the following equation.

$$V_n = \frac{DM_n}{t_n - t_{n-1}}$$

Where V_n is the velocity between 2 consecutive frames. DM_n is equal to distance traveled between 2 consecutive frames and ($t_n - t_{n-1}$) is the difference in time between those 2 consecutive frames.

If the software loses track of the animal, this can confound the measurement. In our videos, this occurred in <1% of the 5,400 frames captured per animal. To overcome this, we performed an averaging interval of 3 consecutive measurements, as recommended by EthoVision. The mean was iteratively calculated for 3 consecutive velocity measurements (mean of V_1, V_2, and V_3; mean of V_2, V_3, and V_4…), yielding ~1800 data points (1 data point per 100 milliseconds). These data were then used to calculate the mean velocity during the 3 minute trial.

Acceleration was determined by dividing the difference in velocity measurements between 2 consecutive frames, by the time difference between those frames. Acceleration can be positive or negative, we calculated the absolute acceleration using the following equation:

$$A_n = \frac{V_n - V_{n-1}}{t_n - t_{n-1}}$$

Where A_n is equal to acceleration between 2 consecutive frames. V_n is equal to velocity at frame n, V_{n-1} is equal to the velocity at frame n-1, and ($t_n - t_{n-1}$) is the difference in time between 2 consecutive frames (in milliseconds).

From these A_n data, the mean acceleration per second was calculated and reported.

Turn angle (TA) is the change in angle of the center point of the animal between 3 frames. We calculated the absolute turn angle and did not assess the direction of the turn angle (i.e. clockwise or counterclockwise). The calculation was based on the change in Heading (HE) of the center body point between frames n-2, n-1, and n. From these data HE1 and HE2 angle can be calculated, as shown in the diagram below. Similar to the velocity analysis, we performed an averaging interval of 3 consecutive measurements. This generates the TA every 0.1 seconds (~1,800 data points). These data were used to calculate the mean TA during the 3 minute trial.

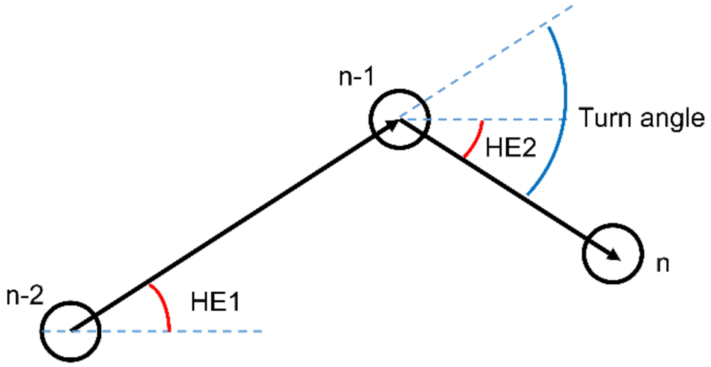

Angular velocity is the turn angle per second. We calculated the absolute angular velocity using the turn angle data generated above. The data points and the following equation:

$$AAV_n = \frac{ATA_n}{t_n - t_{n-1}}$$

Where AAV_n is the absolute angular velocity (where n is each 1 second interval during the 3 minute trial), ATA_n is the sum of the absolute turn angles calculated as described above over the designed time window t_n-t_{n-1} (1000 milliseconds).

These 180 data points which were averaged to yield the mean turn angle per second for the 3 minute trial.

Lateral motion is the percent change in area of the animal's body between 2 consecutive frames. It is calculated using a 2-step method. The first step determines the area (pixels) that have changed (CA_n) using the following equation:

$$CA_n = (A_n - A_n \cap A_{n-1}) + (A_{n-1} - A_n \cap A_{n-1})$$

Where CA_n is the changed area between 2 frames, A_n is the area for frame n, and A_{n-1}

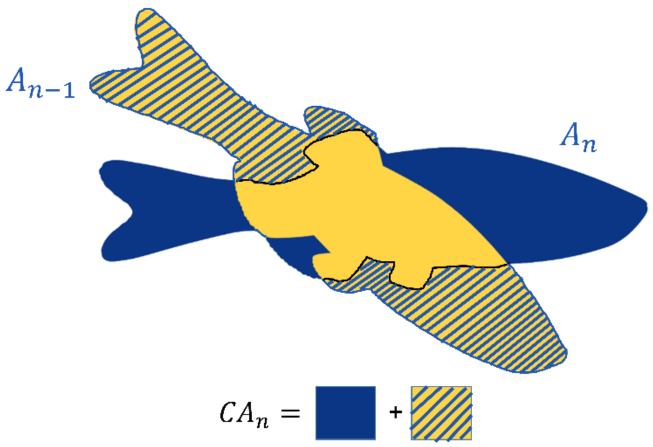

the area for frame n–1.

Next, lateral motion is calculated using the following equation:

$$Mobility = \frac{CA_n}{A_{n-1} + A_n} x\ 100$$

SUPPLEMENTARY RESULTS

Electrical impedance myography detects altered phase angle, reactance, and resistance in the caudal muscles of aged *Tübingen* zebrafish. We conducted impedance measurements in a second cohort of young and aged animals, and demonstrated similar findings. Here, we used the *Tübingen* strain. This is relevant because, in addition to the *casper* strain, many transgenic lines are generated in the *Tübingen* strain. Thus, we sought to confirm the age effect on impedance measurements in the *Tübingen* strain.

EIM across a range of frequencies (1 kHz – 1 MHz) was measured in the caudal muscles of young (4 months: n=4 females and n=4 males) and aged (24 months: n=4 females and n=4 males) zebrafish. In aged animals, there was an overall trend toward decreased phase and reactance at low frequencies as compared to young animals (**Supplementary Figure 4A-C**). Single frequency 2 kHz analyses in aged animals showed significantly decreased phase angle (8.6 ± 3.2 versus 16.7 ± 3.7 kHz; p=0.002, FDR ≤ 0.05) and reactance (148.7 ± 94.2 versus 372.6 ± 157.9 kHz; p=0.005, FDR ≤ 0.05), while resistance was normal as compared to young animals (**Supplementary Figure 4D-F**). At frequencies ≥50 kHz, there were no significant differences (**Supplementary Figure 4G-L)**. Cole parameters were also calculated at low and high frequencies (Supplementary Tables 3-4).

We also determined the reproducibility of EIM data measurements between repeated measures in the same animal (**Supplementary Figure 4M)**. To accomplish this, in nine animals, the needle electrode was inserted and 4 serial measurements were captured. Next, the electrode was removed and then reinserted into the skeletal muscle in the same location (as best as was possible) in order to capture 4 additional serial measurements in the same animal. The measurements recorded by the first and second insertions were highly concordant (**Supplementary Figure 4M)**. The mean percent difference between these two sets of measurements was 9.2 ± 2.1 %. When comparing the reproducibility of the first needle insertion (i.e. the 4 serial measurements), the mean percent difference was 2.9 ± 0.5 %. When comparing the reproducibility of second needle insertion (i.e. the 4 serial tests) the mean percent difference was 3.0 ± 0.6 %.

Age-related changes in swimming performance correlate with electrical impedance myography measurements in *Tübingen* zebrafish. In a second cohort, aged zebrafish exhibited decreased total distance traveled (1073 ± 152 versus 1439 ± 300 cm; p=0.02), decreased velocity (6.00 ± 0.88 versus 8.27 ± 1.59 cm/s; p=0.009), and decreased acceleration (11.8 ± 1.42 versus 18.4 ± 4.18 cm/s^2; p=0.0001) as compared to young zebrafish (**Supplementary Figure 5A-C**; all FDR ≤ 0.05). Young zebrafish were capable of body maneuvers that resulted in deeper turn angles as compared to aged fish (10.56 ± 2.73 versus 6.94 ± 1.77 degrees, p=0.02; **Supplementary Figure 5D**; FDR ≤ 0.05). In addition, young fish exhibited greater angular velocity as compared to aged fish, indicating that it takes more time for aged fish to change their direction of travel (52.00 ± 8.06 versus 36.52 ± 9.71 degrees/s, p=0.03; **Supplementary Figure 5E**; FDR ≤ 0.05). Lastly, aged zebrafish exhibited decreased lateral motion, i.e. non-displacement motion, as compared to young zebrafish (68.01 ± 13.80 versus 94.99 ± 26.96 %, p=0.005; **Supplementary Figure 5F**; FDR ≤ 0.05). Thus, in a second cohort, aged zebrafish exhibit defects in stereotypic parameters of swimming performance as compared to young zebrafish which result from weak, short-duration movements in aged fish as compared to fast, vigorous movements in young fish. Moreover, including both the old and young animals together, there were correlations between 2 kHz phase and every measurement of locomotor function (**Supplementary Figure 5G-L**). Consistent with the first cohort, the strongest correlation with 2 kHz phase was for lateral motion (r=0.8182, p=0.0299, **Supplementary Figure 5L**).

Sample size effects. As this was the first study in zebrafish, our sample size decisions (**Figure 3** and **Supplementary Figure 4**) were guided by our prior EIM studies in other organisms [1-3]. To determine if the sample size we used in **Supplementary Figure 4** and **Figure 3** (in the manuscript) was sufficiently powered to detect EIM changes during aging in zebrafish, we doubled the sample size of the groups as compared to **Supplementary Figure 4** and **Figure 3** (in the manuscript). This revealed the exact same significant findings between groups (**Supplementary Figure 7;** n=17). In the larger

cohort, we found that 2 kHz phase and 2 kHz reactance were significantly different between young and aged zebrafish (**Supplementary Figure 7**) which was also found using smaller cohorts (**Supplementary Figure 4** and **Figure 3**). Importantly, we did not detect any additional differences at other frequencies in the larger cohort (**Supplementary Figure 7F-L**). These findings indicate that a sample size of 7-8 is sufficiently powered to detect age-related defects in zebrafish.

SUPPLEMENTARY FIGURES

A

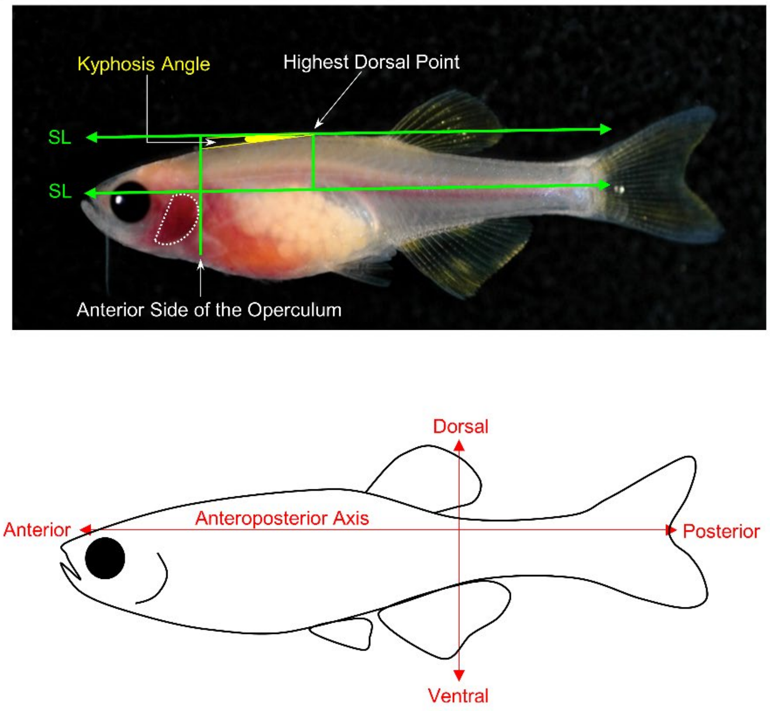

Supplementary Figure 1. Calculating kyphosis angle. A) At the highest dorsal point of the outward curvature of the spine, a line was drawn that was parallel to the standard-length line (green lines). Next, a line was drawn at the anterior side of the operculum (outlined with a white dotted line). The angle between the green line and the dorsal caudal musculature was measured in ImageJ. **B)** Diagram of anteroposterior and dorsoventral axes.

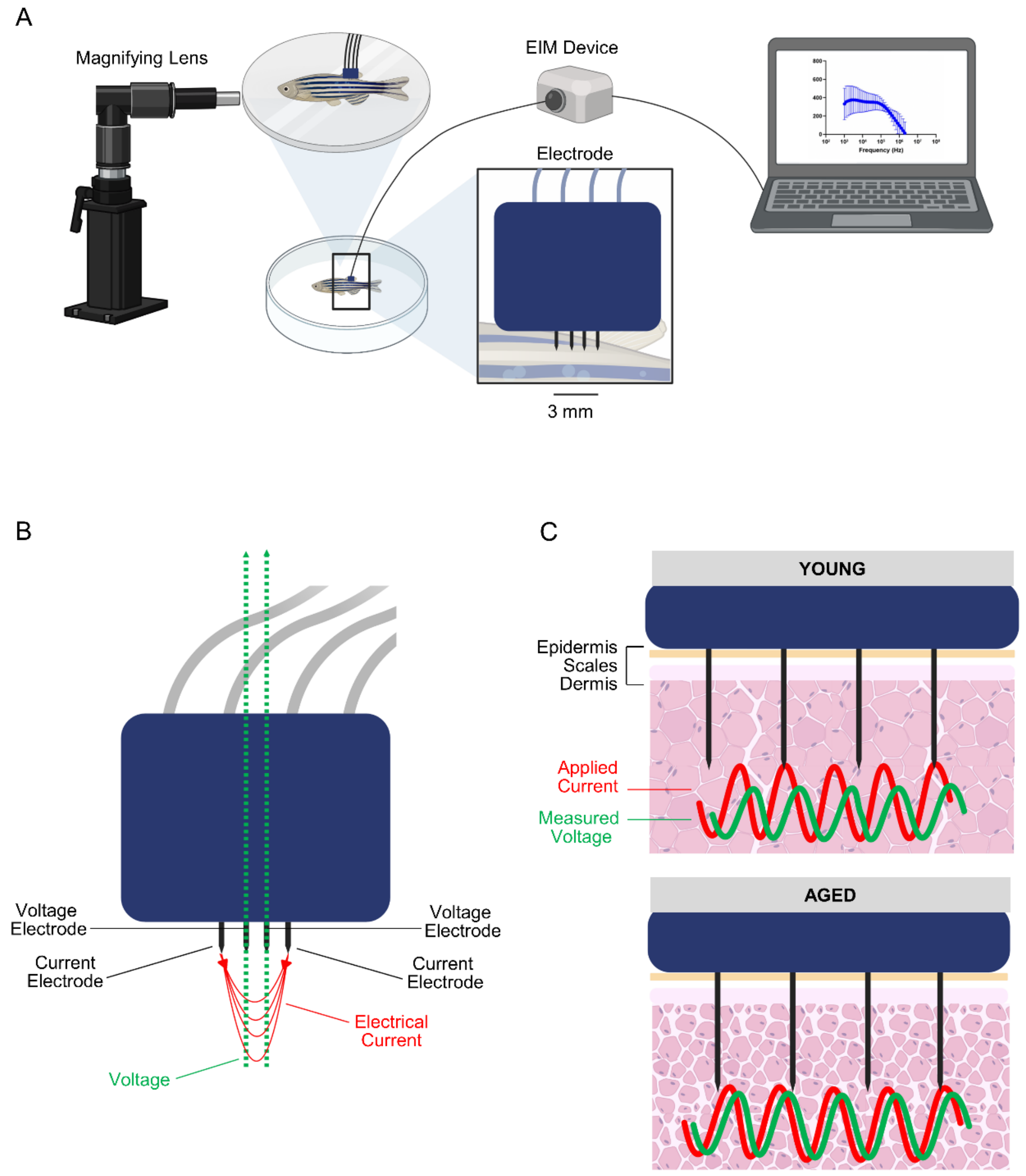

Supplementary Figure 2. Electrical impedance myography in zebrafish: Experimental set-up. A) Diagram of the experimental procedure. **B)** A low-amplitude, high-frequency alternating electrical current is applied to the muscle through a pair of electrodes, and the resulting voltages are measured via a second pair of electrodes. The measurement is completed in <1 minute. **C)** The 1 mm needle array punctures through the epidermis, layered calcified scales, and dermis (together this is ~100 μM thick), and then enters the skeletal muscle. Cartoon depiction of zebrafish caudal muscle and changes in measured voltage in young versus aged muscle (note these drawings are not to scale). Compositional and structural features of the muscle impact the resultant voltages, from which the impedance characteristics can be derived. This figure was created with BioRender.com.

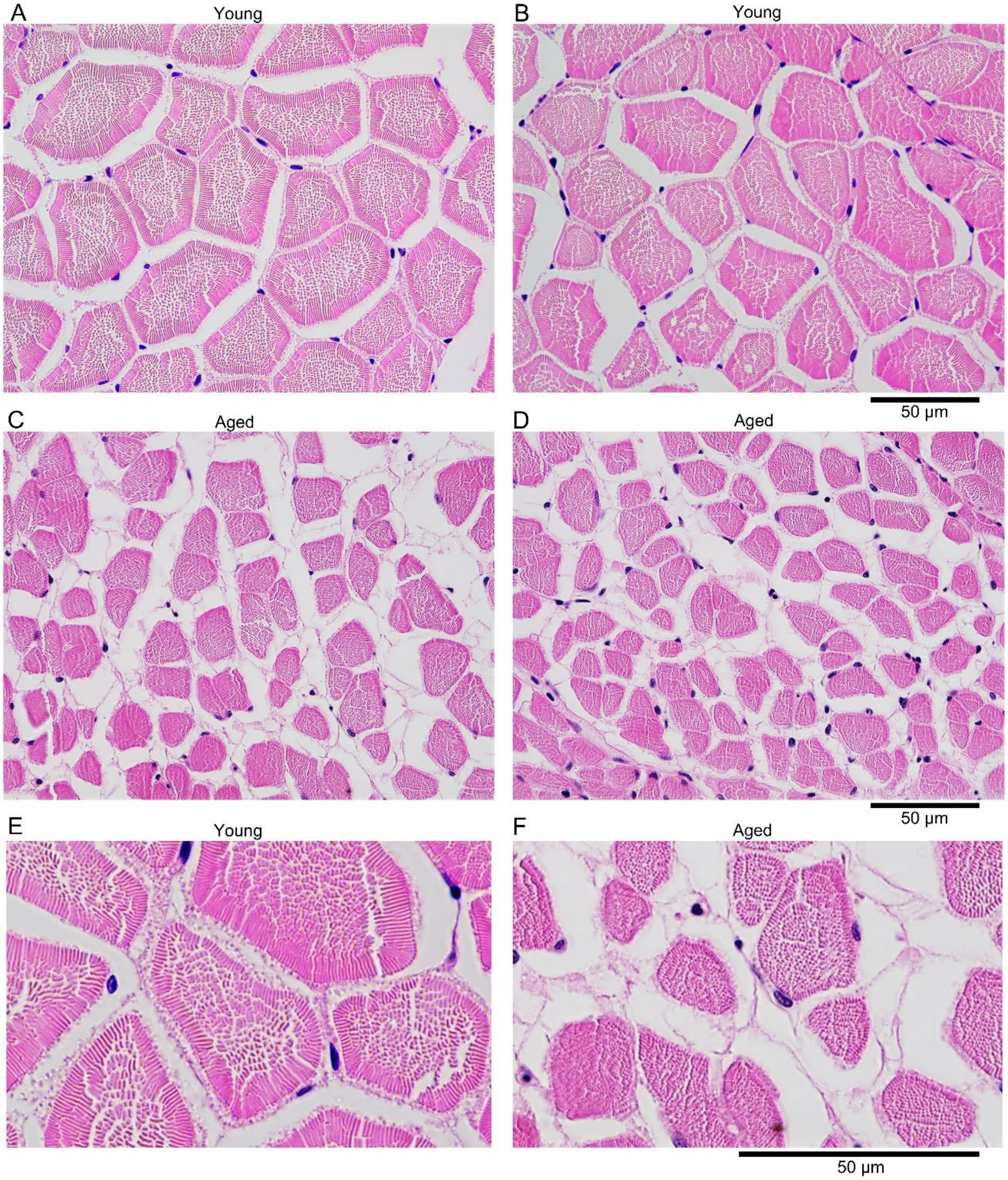

Supplementary Figure 3. Age-related atrophy of skeletal muscle myofibers in *casper* zebrafish. High magnification (60x objective) images of H&E sections from the caudal musculature (distal to the location of the electrode array) of **A,B,E)** young and **C,D,F)** aged animals. Skeletal muscle images of young zebrafish exhibited large muscle cells surrounded by an orderly endomysium (the layer of connective tissue ensheathing the individual muscle fibers), while aged zebrafish exhibited small muscle fibers, disordered endomysium, and increased extracellular spacing between muscle fibers due to atrophy of individual muscle fibers.

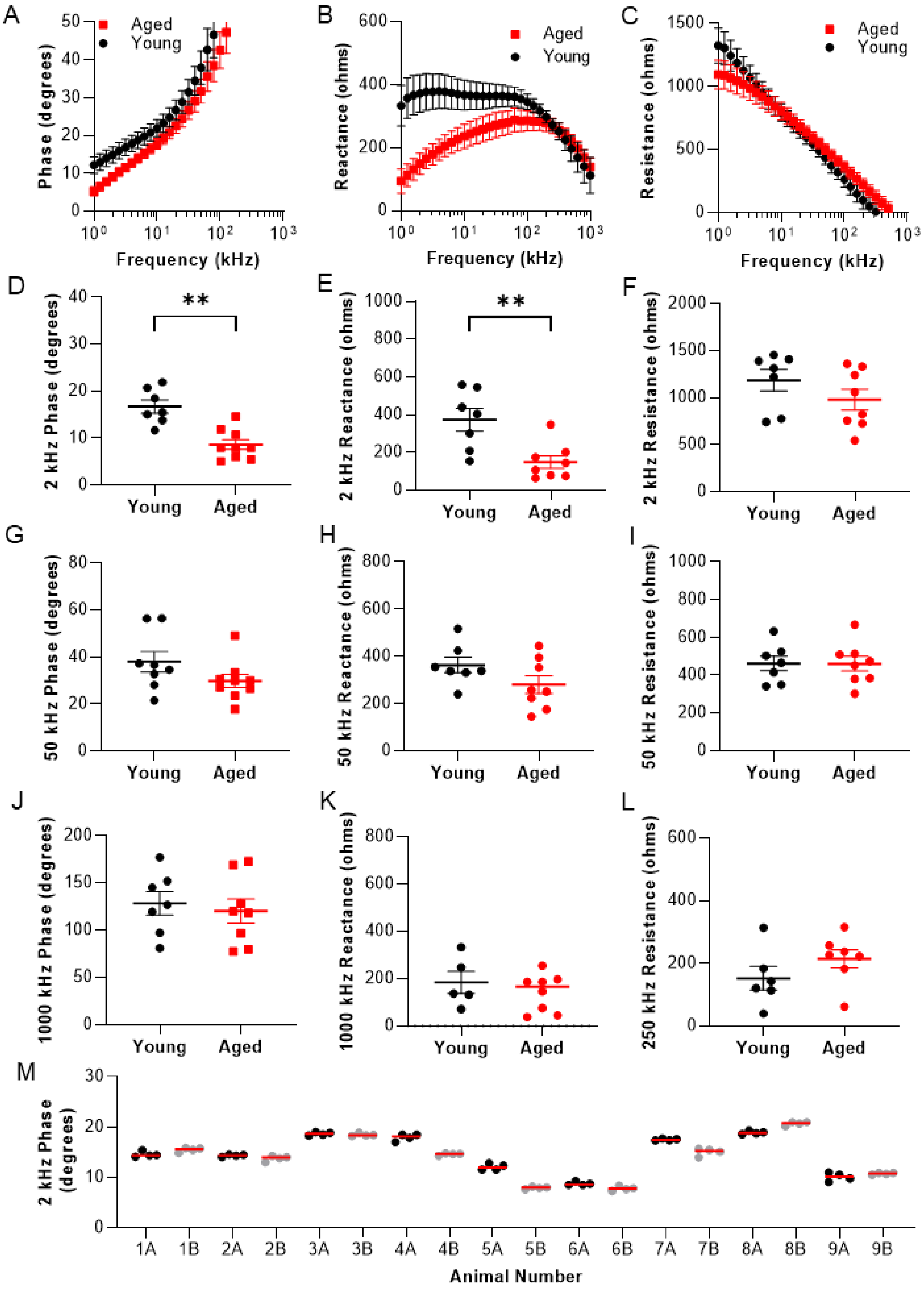

Supplementary Figure 4. Electrical impedance myography detects age-related muscle changes in *Tübingen* zebrafish. Multifrequency graphs (1 kHz – 1 MHz) for **A)** phase, **B)** reactance and **C)** resistance. Single frequency analyses at **D)** 2 kHz phase (q=0.0171), **E)** 2 kHz reactance (q=0.0217), **F)** 2 kHz resistance, **G)** 50 kHz phase, **H)** 50 kHz reactance, **I)** 50 kHz resistance, **J)** 1000 kHz phase, **K)** 1000 kHz reactance, **L)** 250 kHz resistance (n=8) in a second cohort. Data are presented as mean ± SEM. **M)** To assess reproducibility, the electrode was inserted, 4 serial measurements were taken, the electrode was removed and reinserted, and then 4 additional serial measurements were taken. Black circles = measurements 1-4; Gray circles = measurements 5-8; Red bar = mean.

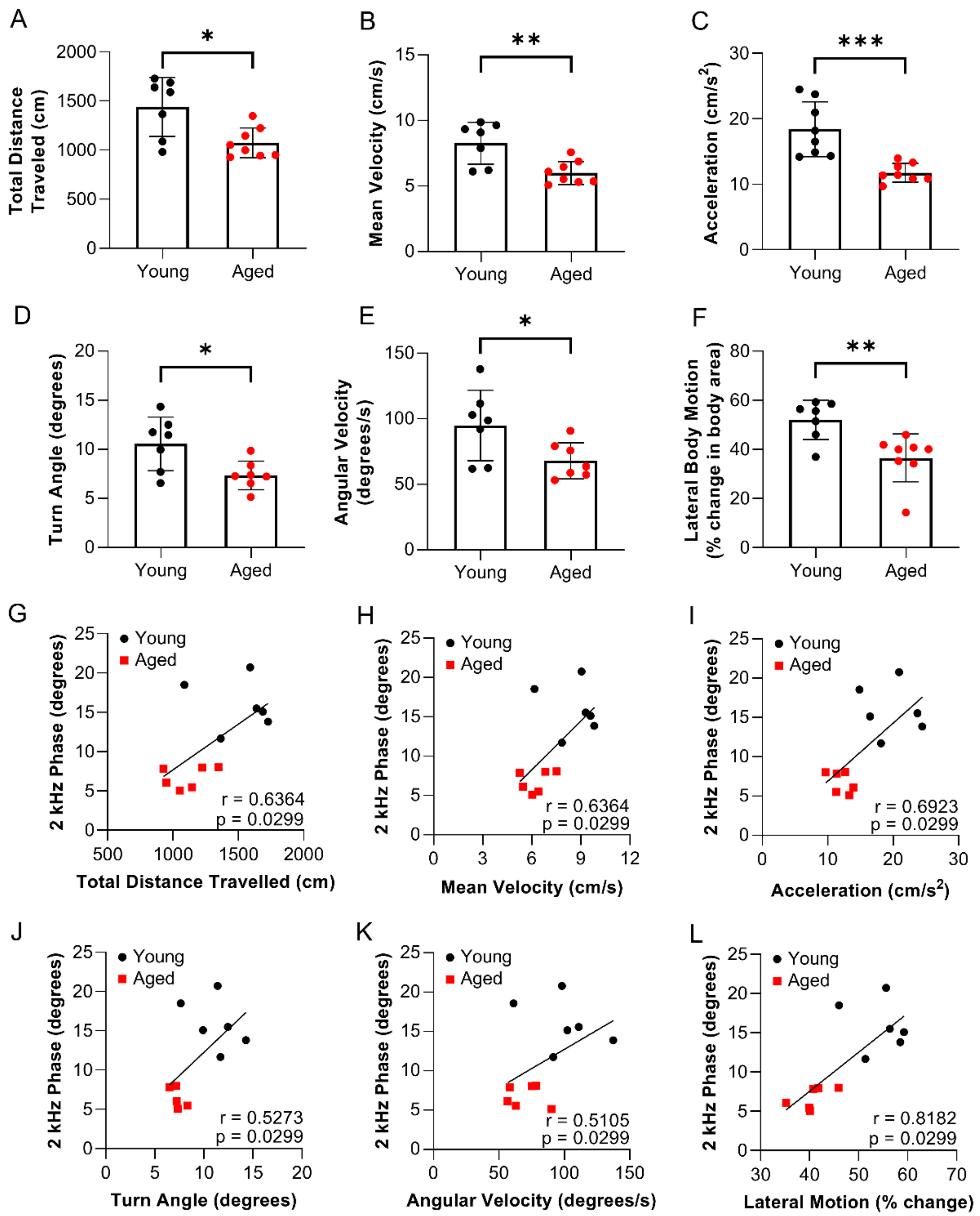

Supplementary Figure 5. Swimming performance deficits in aged animals correlate with electrical impedance myography measurements of caudal muscle resistance in *Tübingen* zebrafish. Locomotor measurements of **A)** total distance traveled (q=0.0323), **B)** velocity (q=0.0195), **C)** acceleration (q=0.0009), **D)** turn angle (q=0.0330), **E)** absolute angular velocity (q=0.0397), and **F)** lateral body motion (q=0.0186) (n=7-8) in a second cohort. Correlation between 2 kHz phase values and **G)** total distance traveled, **H)** velocity, **I)** acceleration, **J)** turn angle, **K)** angular velocity, and **L)** lateral motion for young and aged zebrafish data combined. Spearman correlation r and p values are shown in each panel.

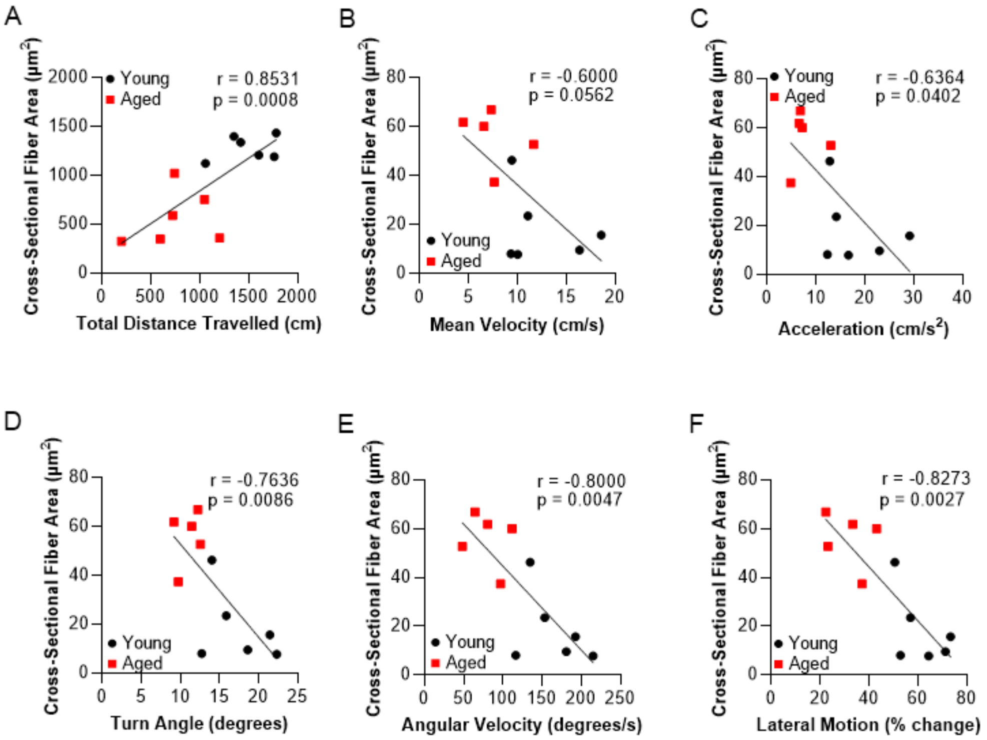

Supplementary Figure 6. Swimming performance deficits in aged animals correlate with age-related changes in muscle morphology in *Tübingen* zebrafish. Correlation between cross-sectional fiber area and locomotor measurements of **A)** total distance traveled, **B)** velocity, **C)** acceleration, **D)** turn angle, **E)** absolute angular velocity, and **F)** lateral body motion. Spearman correlation r and p values are shown in each panel.

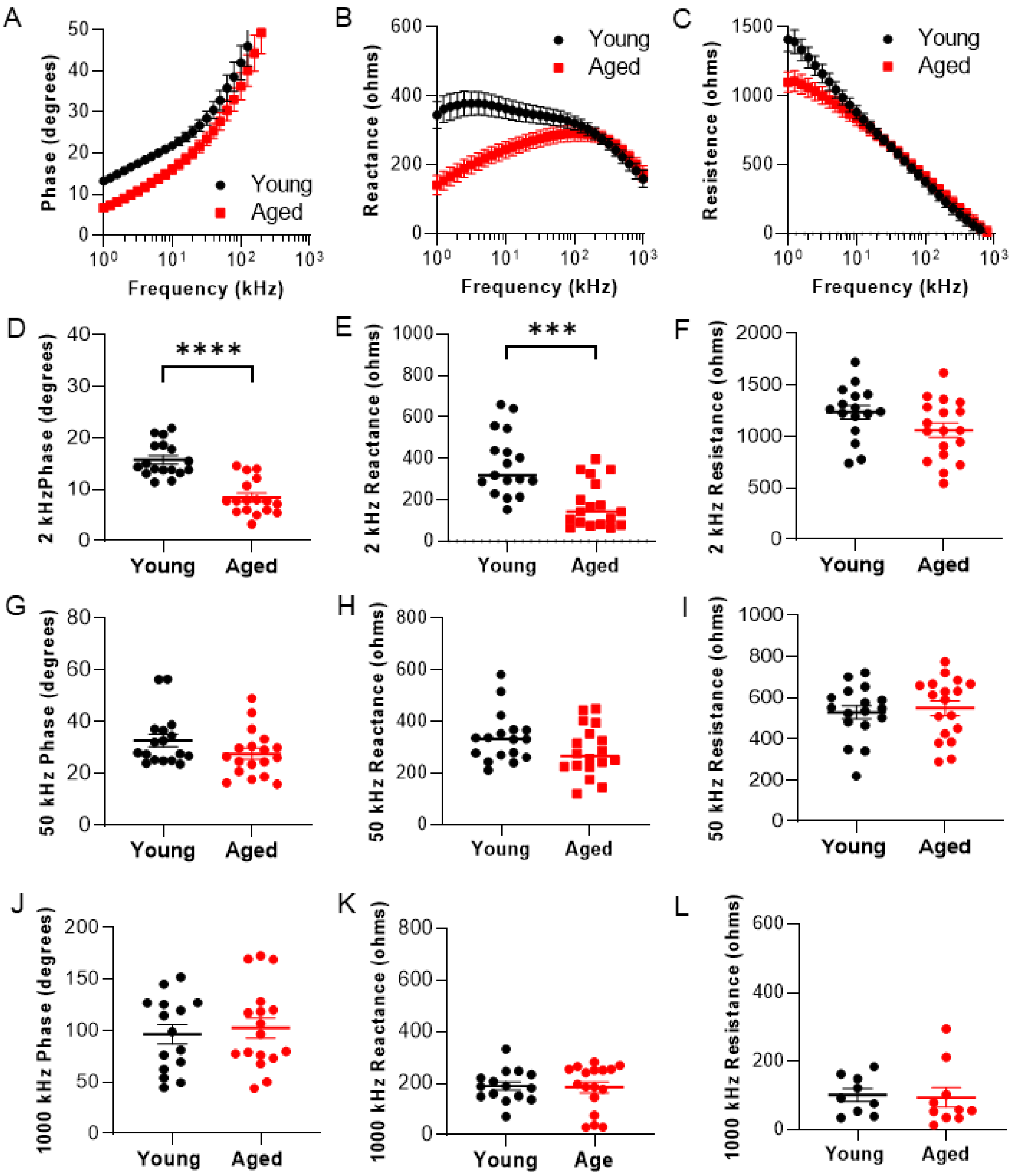

Supplementary Figure 7. Increasing the sample size of the replication cohort and its impact on detecting aged-related changes in EIM parameters. To determine if the sample size in Supplementary Figure 4 and Figure 3 (in the manuscript) was sufficiently powered to detect EIM changes during aging in zebrafish, we doubled the sample size of the groups as compared to Supplementary Figure 4 and Figure 3 (in the manuscript). We found the exact same significant findings between groups, i.e. 2 kHz phase and 2 kHz reactance. Multifrequency graphs (1 kHz – 1 MHz) for **A)** phase, **B)** reactance and **C)** resistance. Single frequency analyses at **D)** 2 kHz phase (q=0.0171), **E)** 2 kHz reactance (q=0.0217), **F)** 2 kHz resistance, **G)** 50 kHz phase, **H)** 50 kHz reactance, **I)** 50 kHz resistance, **J)** 1000 kHz phase, **K)** 1000 kHz reactance, **L)** 1000 kHz resistance (n=17 *Tübingen* zebrafish) in a second cohort. Data are presented as mean ± SEM.

A

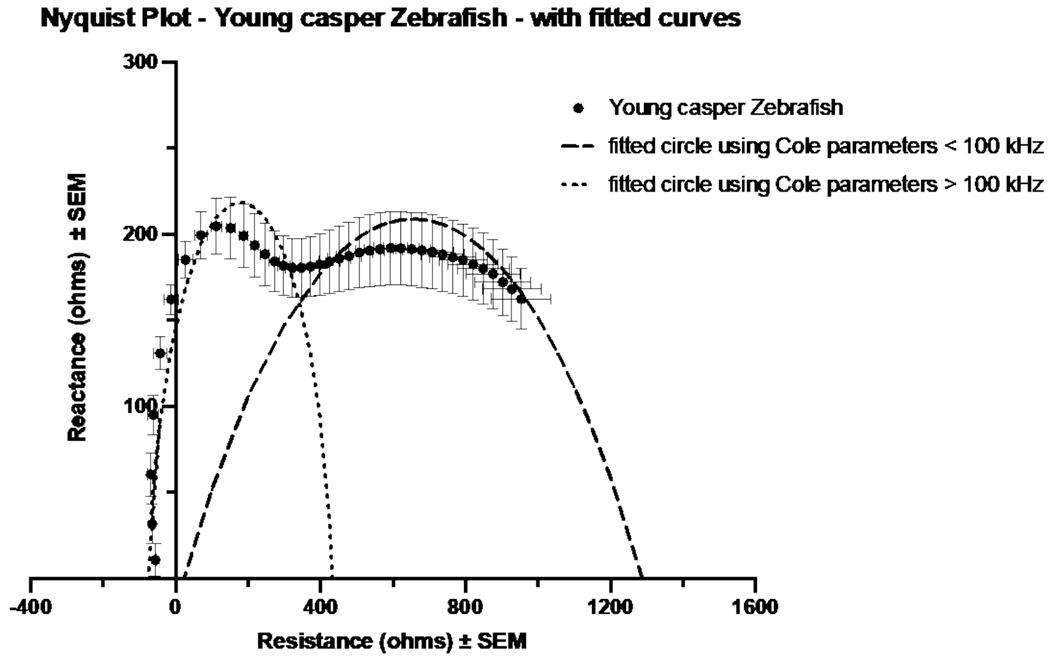

B

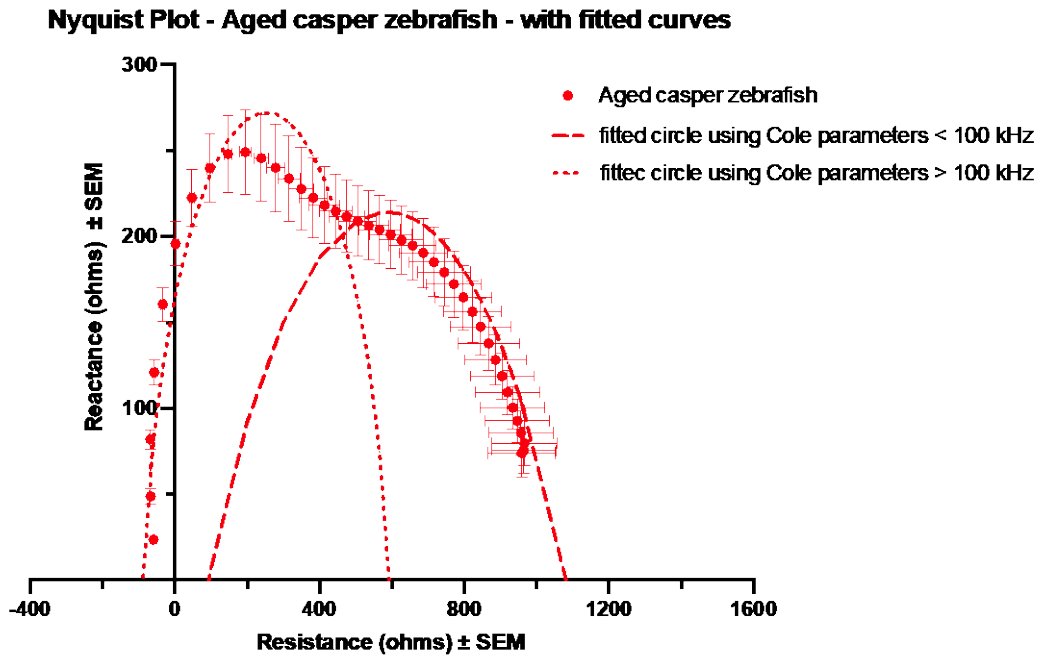

Supplementary Figure 8. Nyquist plots for young and aged *casper* zebrafish. Graphs showing the real (= Resistance) versus the imaginary (= Reactance) part of the complex impedance spectra for young **A)** and aged **B)** *casper* zebrafish. Mean values and standard errors are shown. The dashed and dotted lines represent the Cole model fits generated using the Cole parameters found in Supplementary Tables 1 and 2 corresponding to the respective low (< 100 kHz) and high (> 100 kHz) arcs that are observed in the Nyquist plots for both the young and aged *casper* zebrafish.

Supplementary Table 1. Low frequency Cole parameters in young and aged *casper* zebrafish.

	Cole Parameter[a]					
	R_0 (ohms)	R_∞ (ohms)	f_c (kHz)	alpha	R_0-R_∞ (ohms)	R_0/R_∞
Young	1288 ± 101	23.4 ± 60.8	18.4 ± 6.1	0.406 ± 0.039	1265 ± 116	12.4 ± 18.2
Aged	1081 ± 87	93.5 ± 95.6	70.4 ± 14.8	0.521 ± 0.072	988 ± 112	2.2 ± 2.4
p-Value[b]	0.1797	0.5887	0.0087[c]	0.3939	0.1320	0.9307
Significance	n.s.	n.s.	yes	n.s.	n.s.	n.s.

R_0: resistance at zero frequency; R_∞: resistance at infinite frequency; f_c: center frequency; alpha: a measure of cell size distribution; R_0-R_∞: resistance at zero frequency minus resistance at infinite frequency; R_0/R_∞: the ratio of resistance at zero frequency to resistance at infinite frequency; R_0-R_∞: resistance at zero frequency minus resistance at infinite frequency; kHz: kilohertz; n.s., not significant. Data presented as mean ± standard error of the mean.

[a]Cole parameters were determined for impedance values below 100 kHz.

[b]p-Values were determined using Mann-Whitney tests with an FDR≤0.05.

[c]q=0.0181

[d]The impedance was fit to the complex impedance model described by Cole [4].

$$Z = R_\infty + \frac{R_0 - R_\infty}{1 + (j\omega/\omega_c)^{\alpha}}$$

Where Z is the impedance, R_∞ is the Resistance at infinite frequency, R_0 is the Resistance at zero frequency, ω is the (angular) frequency *in rad* s^{-1} *and* j is the imaginary number $\sqrt{-1}$. The central (angular) frequency, ω_c corresponds to the frequency with the highest absolute value of the imaginary part of the impedance, i.e, the Reactance. The dimensionless parameter α has a value between 0 and 1 and is related to the dispersion of the shape and size distribution.

Supplementary Table 2. High frequency Cole parameters in young and aged *casper* zebrafish.

	Cole Parameter[a]					
	R_0 (ohms)	R_∞ (ohms)	f_c (kHz)	alpha	R_0-R_∞ (ohms)	R_0/R_∞
Young	432±36.5	-75.6±3.98	670±74	0.9055±0.0235	508±39	5.71±0.377
Aged	592±65.2	-88.4±5.32	661±47	0.8597±0.0203	681±70	6.63±0.496
p-Value[b]	0.1014	0.1014	0.5338	0.2949	0.1375	0.1375
Significance	n.s.	n.s.	n.s.	n.s.	n.s.	n.s

R_0: resistance at zero frequency; R_∞: resistance at infinite frequency; f_c: center frequency; alpha: a measure of cell size distribution; R_0-R_∞: resistance at zero frequency minus resistance at infinite frequency; R_0/R_∞: the ratio of resistance at zero frequency to resistance at infinite frequency; R_0-R_∞: resistance at zero frequency minus resistance at infinite frequency; kHz: kilohertz; n.s., not significant. Data presented as mean ± standard error of the mean.

[a]Cole parameters were determined for impedance values above 100 kHz.
[b]p-Values were determined using Mann-Whitney tests.

Supplementary Table 3. Low frequency Cole parameters in young and aged *Tübingen* zebrafish.

	Cole Parameter[a]					
	R_0 (ohms)	R_∞ (ohms)	f_c (kHz)	alpha	R_0-R_∞ (ohms)	R_0/R_∞
Young	2108±252	-135.1±111	6.7±1.2	0.4539±0.0321	2243±332.4	1.388±8.196
Aged	1452±176	-226.2±87.61	60.4±18.9	0.4927±0.0336	1678±232.2	1.716±4.352
p-Value[b]	0.0224[c]	0.2968	< 0.0001[d]	0.3299	0.0814	>0.9999
Significance	yes	n.s.	Yes	n.s.	n.s.	n.s.

R_0: resistance at zero frequency; R_∞: resistance at infinite frequency; f_c: center frequency; alpha: a measure of cell size distribution; R_0-R_∞: resistance at zero frequency minus resistance at infinite frequency; R_0/R_∞: the ratio of resistance at zero frequency to resistance at infinite frequency; R_0-R_∞: resistance at zero frequency minus resistance at infinite frequency; kHz: kilohertz; n.s., not significant. Data presented as mean ± standard error of the mean.

[a]Cole parameters were determined for impedance values above 100 kHz.

[b]p-Values were determined using Mann-Whitney tests with an FDR≤0.05.

[c]q=0.0235

[d]q=0.000021

Supplementary Table 4. High frequency Cole parameters in young and aged *Tübingen* zebrafish.

	Cole Parameter[a]					
	R_0 (ohms)	R_∞ (ohms)	f_c (kHz)	alpha	R_0-R_∞ (ohms)	R_0/R_∞
Young	407.7±41.5	-91.16±15.87	480±173	0.7948±0.1409	498.9±33.24	6.06±0.7875
Aged	407.1±38.2	-96.92±11.66	379±49	0.8133±0.0267	504.0±32.63	5.408±0.708
p-Value[b]	>0.9999	0.2997	0.6526	0.9252	>0.9999	0.5516
Significance	n.s.	n.s.	n.s.	n.s.	n.s	n.s

R_0: resistance at zero; R_∞: resistance at infinite frequency; f_c: center frequency; alpha: a measure of cell size distribution; R_0-R_∞: resistance at zero frequency minus resistance at infinite frequency; R_0/R_∞: the ratio of resistance at zero frequency to resistance at infinite frequency; R_0-R_∞: resistance at zero frequency minus resistance at infinite frequency; kHz: kilohertz; n.s., not significant.
Data presented as mean ± standard error of the mean.

[a]Cole parameters were determined for impedance values above 100 kHz.

[b]p-Values were determined using Mann-Whitney tests.

Supplementary Table 5. Reproducibility of EIM parameters at various frequencies.

Frequency (kHz)	EIM Parameter[a]		
	Phase (degrees)	Reactance (ohms)	Resistance (ohms)
2	5.34±1.17	6.25±1.12	2.95±0.43
50	1.30±0.38	1.43±0.42	1.32±0.28
100	1.05±0.19	1.33±0.37	1.60±0.39

kHz: kilohertz

[a] Reproducibility was assessed by calculating the mean percent difference

SUPPLEMENTARY REFERENCES

1 Sung, M. *et al.* Spaceflight and hind limb unloading induce similar changes in electrical impedance characteristics of mouse gastrocnemius muscle. *J Musculoskelet Neuronal Interact* **13**, 405-411 (2013).

2 Li, J., Pacheck, A., Sanchez, B. & Rutkove, S. B. Single and modeled multifrequency electrical impedance myography parameters and their relationship to force production in the ALS SOD1G93A mouse. *Amyotroph Lateral Scler Frontotemporal Degener* **17**, 397-403, doi:10.3109/21678421.2016.1165258 (2016).

3 Li, J., Yim, S., Pacheck, A., Sanchez, B. & Rutkove, S. B. Electrical Impedance Myography to Detect the Effects of Electrical Muscle Stimulation in Wild Type and Mdx Mice. *PLoS One* **11**, e0151415, doi:10.1371/journal.pone.0151415 (2016).

4 Sanchez, B. *et al.* Evaluation of Electrical Impedance as a Biomarker of Myostatin Inhibition in Wild Type and Muscular Dystrophy Mice. *PLoS One* **10**, e0140521, doi:10.1371/journal.pone.0140521 (2015).
